# Enhanced Protein Synthesis and Hippocampus‐Dependent Memory via Inhibition of *YTHDF2*‐Mediated m^6^A mRNA Degradation

**DOI:** 10.1002/advs.202514926

**Published:** 2025-09-17

**Authors:** Kuan Li, Chen Guo, Xiaoli Wu, Cuiting Wu, Songfen Wu, Si Su, Min Wu, Xidan Zhou, Si Li, Yihui Cui, Tao Zhou

**Affiliations:** ^1^ Department of Pharmacy Shenzhen People's Hospital (The Second Clinical Medical College Jinan University The First Affiliated Hospital) Southern University of Science and Technology Shenzhen 518055 China; ^2^ Department of Neurology of Sir Run Run Shaw Hospital and School of Brain Science and Brain Medicine Zhejiang University School of Medicine Hangzhou 310058 China; ^3^ Shenzhen Neher Neural Plasticity Laboratory Shenzhen Key Laboratory of Drug Addiction the Brain Cognition and Brain Disease Institute Shenzhen Institutes of Advanced Technology Chinese Academy of Sciences Shenzhen 518055 China; ^4^ Shenzhen Institutes of Advanced Technology University of Chinese Academy of Sciences Beijing 100049 China; ^5^ Faculty of Life and Health Sciences Shenzhen University of Advanced Technology Shenzhen 518106 China; ^6^ Shenzhen‐Hong Kong Institute of Brain Science‐Shenzhen Fundamental Research Institutions Shenzhen 518055 China

**Keywords:** hippocampus, learning and memory, mRNA degradation, *N* ^6^‐methyladenosine, protein synthesis, synaptic transmission, YHTDF2

## Abstract

*N* ^6^‐methyladenosine (m^6^A) modification intricately regulates mRNA transportation, localization, and translation, significantly influencing learning and memory processes. However, the specific role of YT521‐B homology (YTH) domain‐containing family protein 2 (*YTHDF2)*‐mediated m^6^A mRNA degradation in learning and memory remains elusive. Utilizing a forebrain‐specific conditional knockout mice model, it is discovered that the absence of *YTHDF2* impedes the decay of m^6^A‐modified mRNAs, resulting in heightened synaptic transmission in hippocampal neurons and improved hippocampus‐dependent learning and memory. Unexpectedly, an increase in activity‐dependent protein synthesis is also observed. Reintroduction of *YTHDF2* expression or reduction of its downstream target, Semaphorin 4B (*SEMA4B)*, in the hippocampus reverses the enhanced memory in conditional knockout mice, while augmenting *YTHDF2* in wild‐type mice impairs memory performance. These findings underscore the pivotal role of *YTHDF2*‐mediated mRNA degradation in regulating learning and memory processes.

## Graphical abstract

*YTHDF2*‐mediated m6A mRNA decay critically regulates learning and memory. Its forebrain‐specific knockout in mice impedes degradation of m6A‐modified mRNAs, enhancing hippocampal synaptic transmission, protein synthesis, and memory. Conversely, hippocampal *YTHDF2* reintroduction or *SEMA4B* reduction reverses this enhancement, while its overexpression impairs memory, establishing *YTHDF2*’s pivotal role.

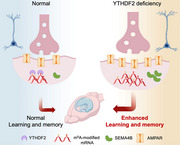

## 1 Introduction

*N* ^6^‐methyladenosine (m^6^A) stands out as the predominant reversible mRNA modification in the brain.^[^ ^1^ ^]^ The effects of m^6^A are orchestrated by its writer, eraser, and reader proteins.^[^ ^2^ ^]^ A multicomponent writer complex, comprising the methytransferase 3 (*METTL3)* catalytic subunit and several accessory subunits, deposits m^6^A. Two demethylases, fat mass and obesity‐associated gene (*FTO)* and AlkB Homolog 5 (*ALKBH5)*, act as erasers.^[^ ^3^ ^]^ The binding of reader proteins to m^6^A‐modified mRNAs influences the metabolism of these transcripts. For example, the reader protein *YTHDF2* promotes m^6^A‐mediated degradation of mRNAs, while *YTHDF1* and *YTHDF3* facilitate m^6^A‐dependent mRNA translation.^[^ ^4^ ^]^ The dynamic nature of m^6^A and its pervasive influence across all facets of mRNA processing have established this epigenetic modification as a pivotal regulator of brain functions, particularly in relation to learning and memory.^[^ ^5^ ^]^ Notably, m^6^A signaling has been shown to govern gene expression across various forms of neural plasticity crucial for learning and memory. Studies have revealed heightened m^6^A levels in the medial prefrontal cortex (mPFC) of mice following learning behavioral experience,^[^ ^6^ ^]^ while molecular adjustments of *METTL3* and *FTO*,^[^ ^4^ ^]^ have been demonstrated to modulate spatial memory and synaptic plasticity.^[^ ^7^ ^]^ Specifically, depletion of *METTL3* in the mouse hippocampus led to deficiencies in hippocampal long‐term potentiation (LTP) and long‐term spatial memory by impacting the translation of early response genes in neurons, whereas overexpression of *METTL3* significantly bolstered memory consolidation.^[^ ^8^ ^]^ In addition to its methyltransferase activity, m^6^A demethylation also proves crucial for memory formation, as evidenced by a transient reduction in *FTO* abundance during memory formation, particularly at the synapse.^[^ ^9^ ^]^ Depletion of *FTO* resulted in enhanced memory in contextual fear conditioning task.^[^ ^9^, ^10^ ^]^ Moreover, our prior research revealed that the deletion of the m^6^A reader *YTHDF1*, which promotes the translation of m^6^A‐modified transcripts, led to memory deficits, impaired hippocampal synaptic transmission, and long‐term potentiation, while reexpression of *YTHDF1* in the hippocampus rectified the behavioral and synaptic deficiencies.^[^ ^11^ ^]^ Collectively, these findings underscore the necessity of m^6^A and its regulation of mRNA translation for optimal learning. However, the involvement of m^6^A‐mediated mRNA degradation in learning and memory, as well as its mechanisms, remains elusive.

*YTHDF2* is involved in the rapid degradation of transcripts through two distinct pathways: deadenylation via recruitment of carbon catabolite repression 4/negative on TATA‐less (CCR4/NOT) complex or endoribonucleolytic cleavage by the *YTHDF2*–heat responsive protein 12 (HRSP12)–ribonuclease (RNase) P/mitochondrial RNA‐processing complex.^[^ ^12^, ^13^, ^14^, ^15^ ^]^ *YTHDF2* is essential for nervous system development. Global *YTHDF2* knockout causes embryonic lethality, while heterozygotes show semilethality, likely due to disrupted neural stem/progenitor cell (NSPC) self‐renewal and impaired neuron/glia generation in the embryonic neocortex.^[^ ^16^ ^]^ Additionally, *YTHDF2*‐mediated transcripts turnover has been identified to be involved in neural stem cell (NSC) renewal and differentiation in the cortex of the *METTL3* and *METTL14* knockout mouse brain.^[^ ^17^ ^]^

The hippocampus, a crucial component of the limbic system in the forebrain, is known to play a pivotal role in learning and memory.^[^ ^18^ ^]^ A recent study indicates that specific ablation of *YTHDF2* in the dentate gyrus (DG) of mice hippocampus caused mossy fiber overgrowth and impairment of mossy fiber‐granule cell (MG‐CA3) synaptic transmission, leading to impaired memory formation, suggesting *YTHDF2*’s involvement in hippocampus‐dependent learning and memory.^[^ ^19^ ^]^ However, the exact function of *YTHDF2* and *YTHDF2*‐mediated mRNA decay in hippocampal function and learning behavior remains poorly understood. While the DG plays significant role in hippocampal memory formation, it is important to note that DG lesion only impairs some, but not all, hippocampus‐dependent memory functions, indicating the indispensable role of the rest of the hippocampus in memory formation.^[^ ^20^ ^]^ We then employed a forebrain‐specific conditional knockout (CKO) mice model to investigate the function of *YTHDF2* in the mice hippocampus. Our findings revealed that the depletion of *YTHDF2* in the entire hippocampus led to enhanced activity‐dependent protein synthesis and improved hippocampus‐dependent memory. These results emphasize the critical role of *YTHDF2* and the detrimental impact of *YTHDF2*‐mediated transcript degradation in regulating learning and memory in the mouse brain.

## 2 Results

### 2.1 Loss of *YTHDF2* in the Forebrain Enhances Hippocampus‐Dependent Learning and Memory

**Figure 1:**
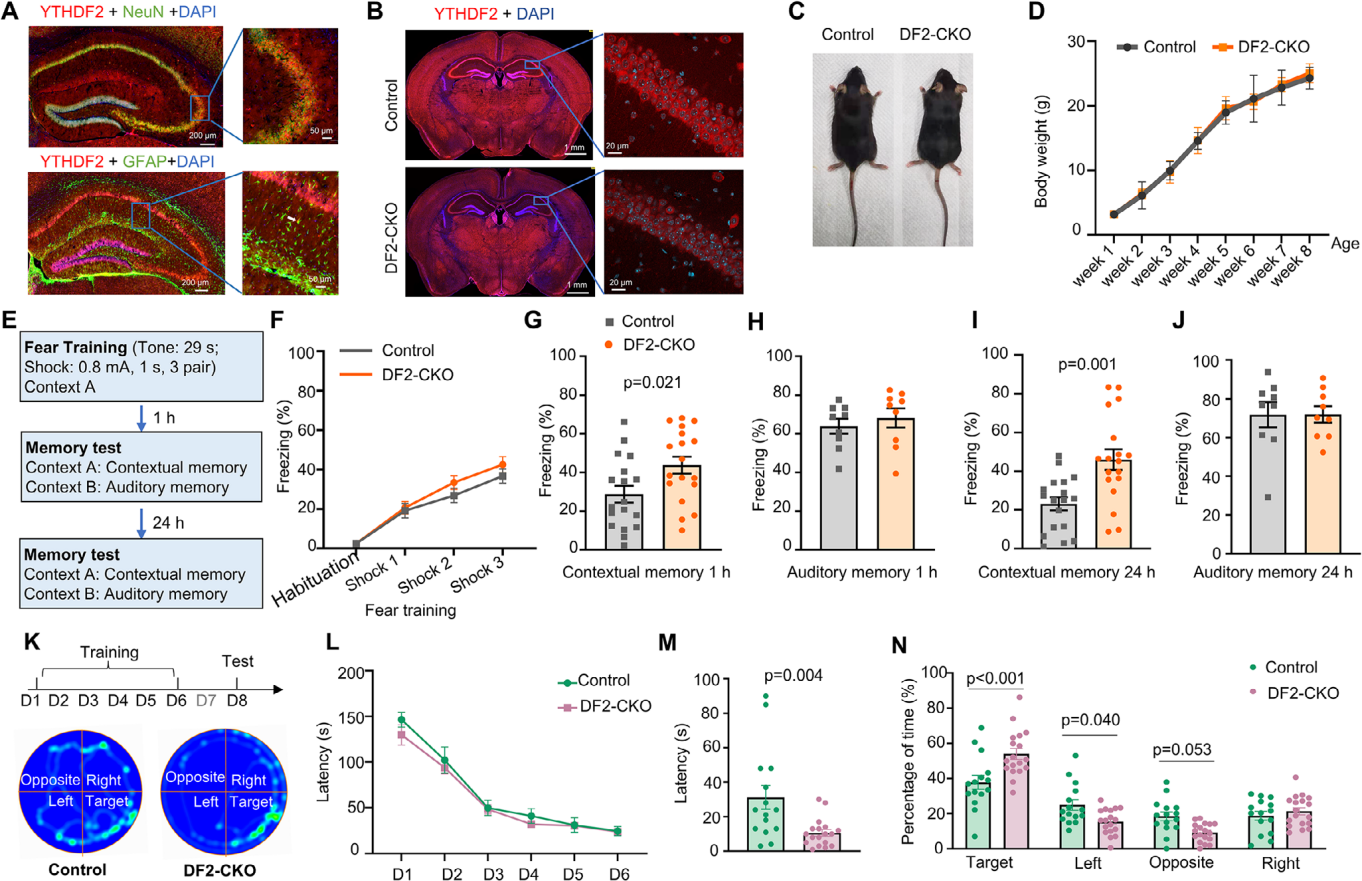
Enhanced learning and memory in DF2‐CKO mice. A) Representative images of co‐immunostaining for *YTHDF2* with NeuN (upper panel) or GFAP (down panel) in the mouse hippocampus. *YTHDF2* predominantly colocalized with the neuronal marker NeuN, and sparsely with the astrocytic marker GFAP. B) Representative immunostaining images of *YTHDF2* in control and DF2‐CKO mice, showing efficient knockout of *YTHDF2* expression in both the hippocampus and cortex. C) Representative images of adult (10 weeks old) control and DF2‐CKO mice. D) Body weight curves of control and DF2‐CKO mice from born to 8 weeks old (*n* = 7 mice per group, two‐way ANOVA, *F*(1, 96) = 0.363, *p* = 0.548), indicating no significant difference in body weight between groups. E) Schematic representation of the protocol used to test fear memory. F) The freezing level curves of control and DF2‐CKO mice during fear conditioning (*n* = 18 mice per group, two‐way ANOVA, Control vs DF2‐CKO: *F*(1, 34) = 1.006, *p* = 0.323). G,H) Contextual (G) and auditory (H) fear memory assessed at 1 h after fear training (G: *n* = 18 mice per group, unpaired two‐tailed *t*‐test, *t*_34_ = 2.422, *p* = 0.021; H: *n* = 9 mice per group, unpaired two‐tailed *t*‐test, *t*_16_ = 0.677, *p* = 0.508). I,J) Contextual (I) and auditory (J) fear memory assessed 24 h after fear training (I: *n* = 18 mice per group, unpaired two‐tailed *t*‐test, *t*_34_ = 3.611, *p* = 0.001; J: *n* = 9 mice per group, unpaired two‐tailed *t*‐test, *t*_16_ = 0.018, *p* = 0.986). K) The Barnes maze test was used to assess spatial memory. The upper panel shows the training protocol, and the lower panel shows heat maps of movement trajectories for control and DF2‐CKO mice on the test day (day 8). L) Performance of control and DF2‐CKO mice in training sessions of Barnes maze test (*n* = 15, 18 mice per group, two‐way ANOVA, group: *F*(1, 186) = 2.026, *p* = 0.156). M) Latencies (time to locate the escape box) in the probe trial of the Barnes Maze test (*n* = 15, 18 mice per group, unpaired *t*‐test, *t*_31_ = 3.101, *p* = 0.004). N) The time spent in target quadrant and other quadrants during the probe trial of the Barnes Maze test (*n* = 15, 18 mice per group, two‐way ANOVA, *F*(3, 124) = 11.1, *p* < 0.001, post hoc: Bonferroni's test, target: *p* < 0.001, left: *p* = 0.040, opposite: *p* = 0.053, right: *p* > 0.999).

Previous reports have identified *YTHDF2* exhibits high expression level in the mouse brain^[^ ^21^ ^]^ and in different cell types, including neuron, glia, and ependymal cell.^[^ ^22^ ^]^ Through immunostaining analysis, we confirmed the presence of *YTHDF2* protein in different cell types in the adult mouse hippocampus. Our findings indicated that *YTHDF2* is prominently expressed in neurons, as it mainly colocalized with NeuN and sparsely colocalized with astrocytes markers glial fibrillary acidic protein (GFAP) (**Figure** **1**A). Given the potential involvement of *YTHDF2* in learning and memory,^[^ ^19^ ^]^ we then aimed to explore the functional role of *YTHDF2* in hippocampus‐dependent learning and memory. To achieve this, we developed a forebrain specific *YTHDF2* knockout mouse model, by crossing *YTHDF2^flox/flox^* mice^[^ ^23^ ^]^ with *Emx1‐Cre* mice^[^ ^24^ ^]^ (Figure S1A,B, Supporting Information). Immunostaining and western blot data demonstrated efficient knockout of *YTHDF2* expression in both hippocampus and mPFC (Figure 1B; Figure S1C, Supporting Information) of *YTHDF2^flox/flox^Emx1‐Cre* mice (referred to as DF2‐CKO) compared to *YTHDF2^flox/flox^* (referred to as Control). The Cre‐recombinase‐dependent deletion of *YTHDF2* was further confirmed by examining RNA sequencing data using the Integrative Genomics Viewer (IGV), which verified the deletion of the fourth exon of *YTHDF2* (Figure S1D, Supporting Information). Remarkably, the DF2‐CKO mice remained viable, fertile, and exhibited normal development into adulthood, wih body weight and brain size comparable to their control littermates (Figure 1C,D; Figure S1E,F, Supporting Information). Additionally, examination of cortical and hippocampal structure in adult mice, as revealed by immunostaining of cortical layer specific marker special AT‐rich sequence‐binding protein 2 (SATB2) and chicken ovalbumin upstream promoter transcription factor (COUP‐TF)‐interacting protein 2 (CTIP2), or NeuN and GFAP in the hippocampus, showed no discernible differences between DF2‐CKO and control mice (Figures S1G and S2A,B, Supporting Information). We further assessed hippocampal cell proliferation at embryonic day 17.5 (E17.5), postnatal day 3 (P3), P14, and P30 using BrdU labeling and Ki67 immunostaining. Quantification of BrdU positive cells at all examined stages revealed no significant differences between DF2‐CKO and control mice (Figure S2C, Supporting Information). Similarly, the number of Ki67⁺ cells in the hippocampus at P3, P14, and P30 also showed no significant differences between the two groups (Figure S2D, Supporting Information), suggesting that hippocampal proliferative activity was not altered by *YTHDF2* deficiency. Collectively, these results indicate that the deletion of *YTHDF2* did not affect the normal development of the forebrain in mice. Furthermore, behavioral assessments using the rotarod test, open field test (OFT), elevated plus maze and light–dark box test all suggested no differences in motor abilities and anxiety‐like behaviors between the control and DF2‐CKO mice (Figure S3A–G, Supporting Information).

However, DF2‐CKO mice exhibited enhanced learning and memory ability. Using classical fear conditioning test (the training protocol outlined in Figure 1E), we found that under a moderate training protocol (0.8 mA, 1 s, 3 pair), the freezing curve during fear learning was similar in both control and DF2‐CKO mice (Figure 1F). However, DF2‐CKO mice exhibited enhanced hippocampus‐dependent contextual memory 1 h after training, and this effect became more pronounced 24 h after training, while the auditory fear memory remained unaffected (Figure 1G–J). Gender differences were also examined, revealing that female mice also displayed significantly increased contextual fear memory 24 h after training (Figure S4A–C, Supporting Information), while no behavioral differences were observed in OFT (Figure S4D,E, Supporting Information).

In addition, we applied two different training protocols, namely, one with 5 conditioned–unconditioned stimuli (CS–US) pairs (0.8 mA, 1 s, 5 pair) and the other with only one CS–US pair (0.8 mA, 1 s, 1 pair), to further investigate the performance of DF2‐CKO mice. Similarly, *YTHDF2* deletion significantly enhanced contextual memory, with the deviation in freezing levels appear to be dependent on the strength of the training intensity (Figure S5A–F, Supporting Information). Specifically, the weaker training protocol resulted in an increase in contextual memory only 24 h after training (Figure S5A, Supporting Information), whereas the stronger training protocol led to increased contextual memory both 1 and 24 h after training, and even long‐term auditory fear memory was significantly increased (Figure S5B–F, Supporting Information).

To further confirm the function of *YTHDF2* in hippocampus‐dependent learning and memory, we performed Barens maze test (Figure 1K–N). Indeed, DF2‐CKO mice spent less time finding the target location (Figure 1M) and spent more time staying in the target quadrant (Figure 1N), indicating enhanced spatial memory, which is a highly specific function of hippocampus.^[^ ^18^ ^]^ Together, these data support the idea that conditional deletion of *YTHDF2* in the mouse forebrain enhanced learning and memory formation in the hippocampus but did not affect the general development of the forebrain.

### 2.2 Loss of *YTHDF2* Increases Synaptic Transmission and Dendritic Spine Density of Hippocampal Neurons

**Figure 2:**
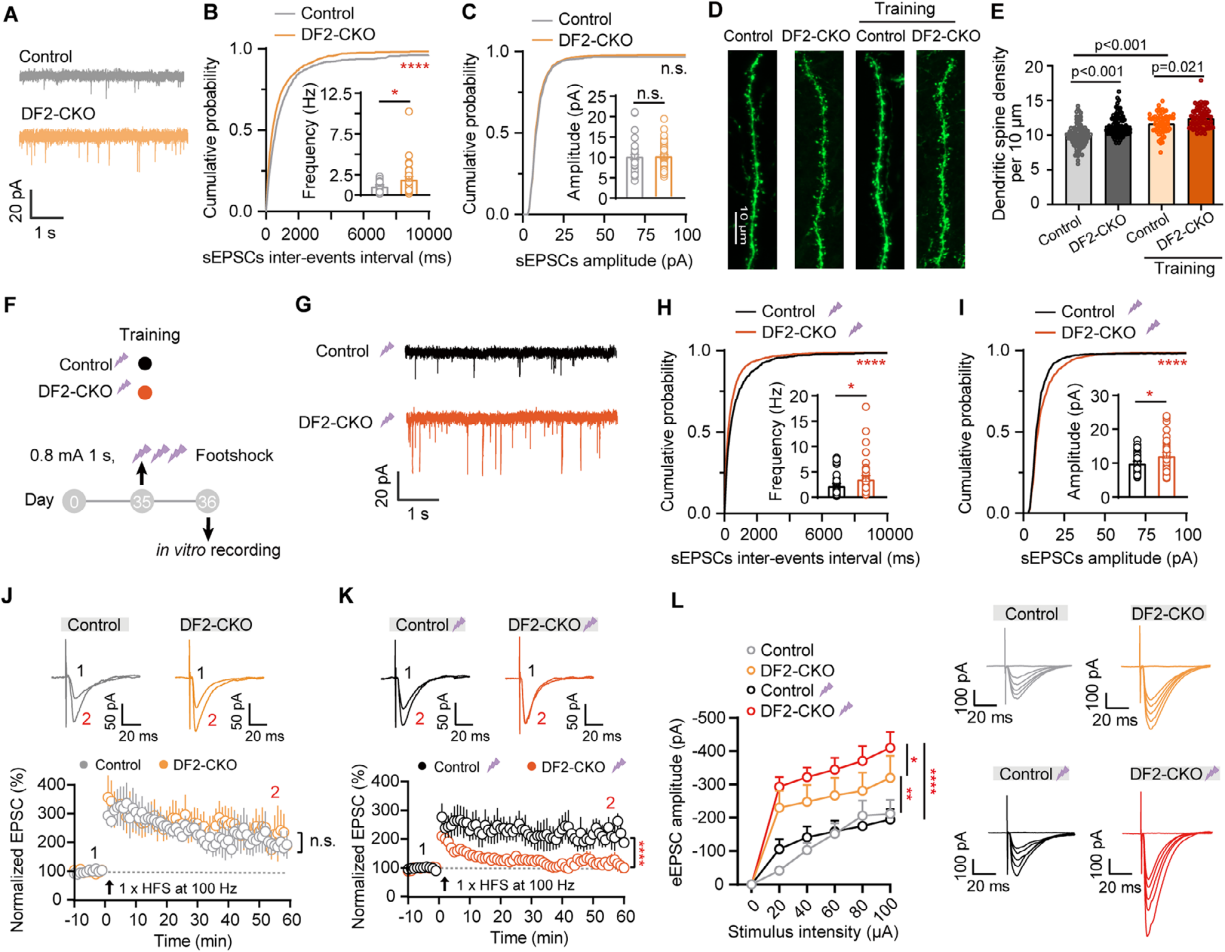
Increased excitatory synaptic transmission and dendritic spine density in DF2‐CKO mice hippocampal neurons. A) Representative traces of spontaneous excitatory postsynaptic currents (sEPSCs) at basal level. B,C) Cumulative probability plots and bar graph (inside) showed an enhanced sEPSCs frequency in DF2‐CKO mice (B) but an unaffected sEPSCs amplitude (C) at basal level. (*n*_control_ = 37 cells, 5 mice, *n*_DF2‐CKO_ = 39 cells, 6 mice; Mann–Whitney test, *U* = 500, *p* = 0.021, KS test, *p* < 0.0001 for frequency; Mann–Whitney test, *U* = 699, *p* = 0.820, KS test, *p* > 0.05 for amplitude). D) Representative images of sparse labeling using NCSP‐YFP‐2E5 showed the morphology of dendrites and spines of a typical CA1 neuron from either control or DF2‐CKO mice at basal condition and 24 h after fear training. E) Statistical analyses of spine density (Control: *n* = 104 dendrites of 9 slices from 3 mice, DF2‐CKO: *n* = 95 dendrites of 12 slices from 3 mice, Control + fear training: *n* = 65 dendrites of 8 slices from 2 mice, DF2‐CKO + fear training: *n* = 73 dendrites of 10 slices from 3 mice; one‐way ANOVA, *F*(3, 333) = 43.53, *p* < 0.001, post hoc: Turkey's test, Control vs DF2‐CKO, *p* < 0.001, Control + training vs DF2‐CKO + training, *p* = 0.021, Control vs Control + training, *p* < 0.001, DF2‐CKO vs DF2‐CKO + training, *p* < 0.001). F,G) Fear conditioning design (F) and representative traces (G) of sEPSCs recorded at 24 h after fear conditioning. H,I) Cumulative probability plots and bar graph (inside) showed both enhanced sEPSC frequency (E) and amplitude (F) in DF2‐CKO mice after fear conditioning. (*n*_control_ = 42 cells, 6 mice, *n*_DF2‐CKO_ = 40 cells, 5 mice; Mann–Whitney test, *U* = 622, *p* = 0.043, KS test, *p* < 0.0001 for frequency; unpaired two‐tailed *t*‐test, *t*_80_ = 2.454, *p* = 0.016, KS test, *p* < 0.0001 for amplitude). J,K) Representative traces (top) and summary plots (bottom) showed LTP induced by high frequency stimulation (HFS) (1 train) at CA3–CA1 pathway from either control or DF2‐CKO mice in naïve condition (J) and after fear conditioning (K) (I: *n*_control_ = 10 cells, 6 mice, *n*_DF2‐CKO_ = 15 cells, 6 mice; unpaired two‐tailed *t*‐test, *t*_18_ = 0.3145, *p* = 0.757; J: *n*_control_ = 9 cells, 5 mice, *n*_DF2‐CKO_ = 13 cells, 4 mice; unpaired *t*‐test, *t*_18_ = 13.79, *p* < 0.0001). L) Representative traces (right) and input–output (I–O) curves (left) showed the amplitudes of evoked EPSC in at CA3–CA1 pathway from either control or DF2‐CKO mice in naïve condition and after fear conditioning (naïve: *n*_control_ = 13 cells from 2 mice, *n*_DF2‐CKO_ = 14 cells from 3 mice; training: *n*_control+training_ = 17 cells from 2 mice, *n*_DF2‐CKO+training_ = 16 cells from 2 mice; one‐way ANOVA, *F*(3, 16) = 19.98, *p* < 0.001, post hoc: Turkey's test, Control vs DF2‐CKO, *p* = 0.004, Control + training vs DF2‐CKO + training, *p* < 0.001, Control vs Control + training, *p* = 0.698, DF2‐CKO vs DF2‐CKO + training, *p* = 0.042). Data are presented as mean ± standard error. **p < 0.01; ***p < 0.001; ****p < 0.0001.

Synaptic plasticity in the hippocampus constitutes the fundamental cellular mechanism for spatial learning and memory. Therefore, we conducted whole‐cell patch clamp recordings in hippocampal CA1 pyramidal neurons to investigate the impact of *YTHDF2* depletion on hippocampal synaptic functions during fear learning. We first measured basal synaptic transmission in hippocampal slices obtained from control or DF2‐CKO mice. Our results showed that DF2‐CKO CA1 pyramidal neurons exhibited an increased frequency of spontaneous excitatory postsynaptic currents (sEPSCs) (**Figure** **2**A–C) under naïve conditions, with no corresponding change in spontaneous inhibitory postsynaptic currents (sIPSCs) (Figure S6A–C, Supporting Information). This suggests a selective enhancement of excitatory synaptic transmission in DF2‐CKO mice. To further investigate presynaptic function, we stimulated the CA3–CA1 pathway while recording from patched CA1 pyramidal neurons (Figure S6D, Supporting Information). The paired‐pulse ratio (PPR) was significantly reduced in DF2‐CKO mice, both at baseline and following trainings (Figure S6E,F, Supporting Information), indicating an elevated presynaptic release probability. Morphologically analysis via sparse labeling revealed increased dendritic spine density in DF2‐CKO CA1 neurons (Figure 2D,E), consistent with the observed synaptic strengthening and suggesting a greater number of excitatory synapses. Notably, this elevated spine density persisted even after moderate fear conditioning training (Figure 2D,E; Figure S6G,H, Supporting Information). 24 h after fear conditioning training, DF2‐CKO mice displayed further enhancement in both the frequency and amplitude of sEPSCs (Figure 2F–I), with no effect on inhibitory transmission (Figure S6I–K, Supporting Information). These findings suggest that fear learning induces additional synaptic potentiation in *YTHDF2*‐deficient CA1 neurons.

Given the enhanced contextual memory observed in DF2‐CKO mice, we then recorded LTP, which is the important cellular model underlying learning and memory, via applying whole‐cell recordings in CA1 neurons. After a stable 10 min of eEPSC baseline recording, one train of high‐frequency stimulation (HFS) was applied at CA3–CA1 synapse to induce LTP. Interestingly, robust LTP was successfully induced in both control group and DF2‐CKO mice with comparable magnitude under naïve conditions (Figure 2J). Given that the synaptic transmission (i.e., increased frequency and amplitude of sEPSCs) were largely enhanced in DF2‐CKO mice after a moderate fear training, we speculate that there is a ceiling effect of LTP during fear learning,^[^ ^25^ ^]^ which may subsequently occlude the HFS‐induced LTP in DF2‐CKO mice after fear training.^[^ ^26^ ^]^ To test this hypothesis, we measured the occurrence and maintenance of LTP in these mice after fear training. As expected, neurons failed to maintain LTP after a 10 min of short‐term potentiation (Figure 2K). Moreover, we recorded the input–output (I–O) curves at different stimulation intensities (0–100 µA). Notably, we found that DF2‐CKO mice showed higher evoked excitatory postsynaptic currents than control mice at basal level. This difference was further amplified following a moderate fear training (Figure 2L), suggesting the deletion of *YTHDF2* facilitates fear learning induced synaptic potentiation in CA1 neurons. Collectively, our data revealed that depletion of *YTHDF2* directly leads to an enhanced excitatory synaptic transmission and facilitates synaptic plasticity of hippocampal neurons, indicating that *YTHDF2* functions as a negative regulator of hippocampus‐dependent learning and memory.

### 2.3 Reexpression of *YTHDF2* in the Adult Hippocampus Reverses the Memory Enhancement Induced by Forebrain *YTHDF2* Deletion

**Figure 3:**
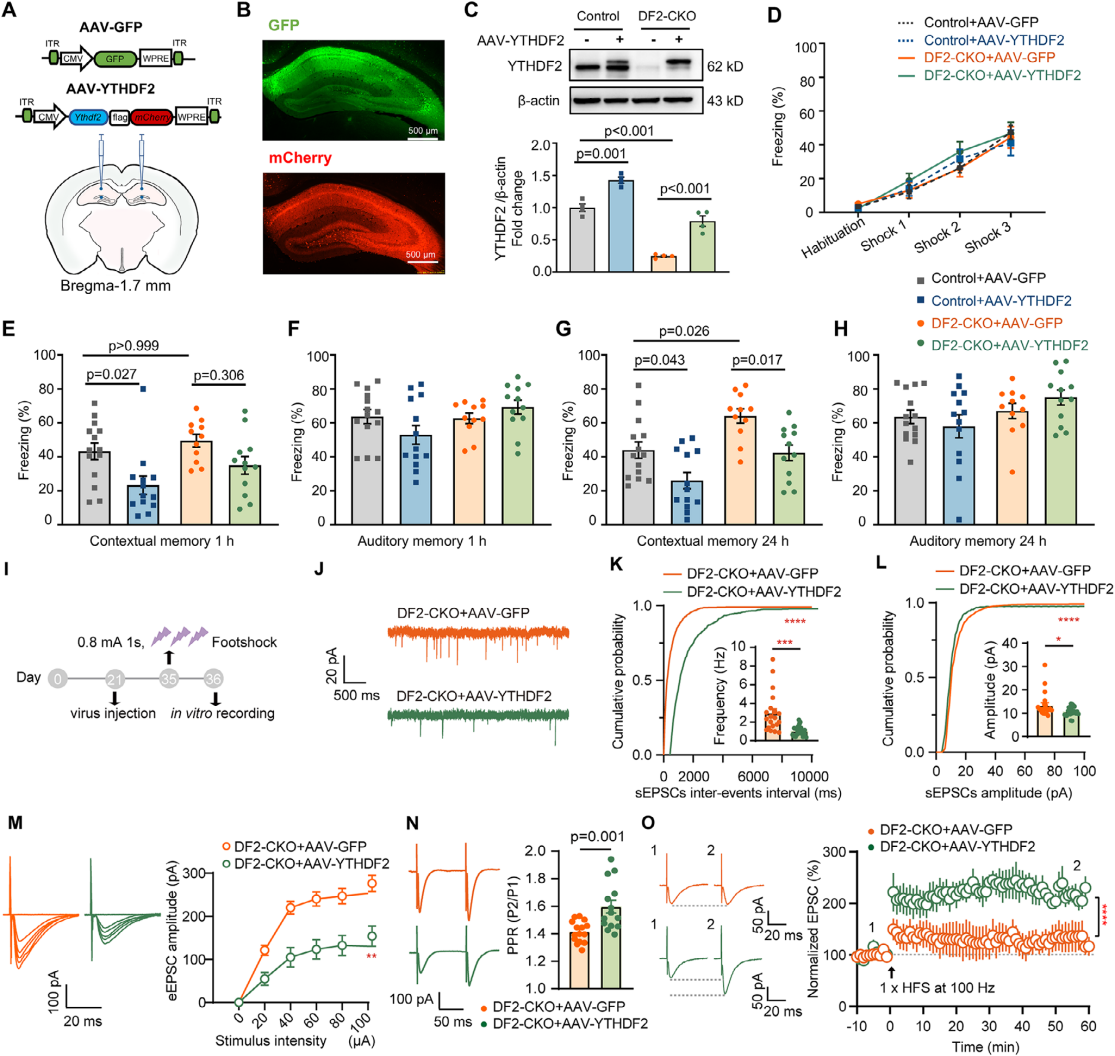
Reexpression of *YTHDF2* in the adult hippocampus reverses the memory performance. A) Schematics of AAV constructs overexpressing *YTHDF2* (AAV–*YTHDF2*) or GFP (AAV–GFP) (up panel) and Illustration of bilateral viral injections into the mouse hippocampus (down panel). ITR, inverted terminal repeats; CMV, cytomegalovirus promoter; WPRE, woodchuck hepatitis virus posttranscriptional regulatory element. B) Representative fluorescence images of the mouse hippocampus after AAV infection. C) Representative western blot (up panel) and quantification data (down panel) of *YTHDF2* expression in mice hippocampus injected with AAV–*YTHDF2* (*n* = 4 mice per group, one‐way ANOVA, *F*(3, 12) = 77.56, *p* < 0.001, post hoc: Bonferroni's test, Control + AAV–GFP vs Control + AAV–*YTHDF2*, *p* = 0.001, DF2‐CKO + AAV–GFP vs DF2‐CKO + AAV–*YTHDF2*, *p* < 0.001, Control + AAV–GFP vs DF2‐CKO + AAV–GFP, *p* < 0.001). D) The freezing curves of control and DF2‐CKO mice injected with AAV during fear conditioning (*n* = 14, 13, 11, 12 mice, two‐way ANOVA, *F*(3, 184) = 0.642, *p* = 0.589). E,F) Contextual (E) and auditory (F) fear memory assessed 1 h after fear training (*n* = 14, 13, 11, 12 mice, E: one‐way ANOVA, *F*(3, 46) = 5.245, *p* = 0.003, post hoc: Bonferroni's test, Control + AAV–GFP vs Control + AAV–*YTHDF2*, *p* = 0.027, DF2‐CKO + AAV–GFP vs DF2‐CKO + AAV–*YTHDF2*, *p* = 0.306, Control + AAV–GFP vs DF2‐CKO + AAV–GFP: *p* > 0.999, Control + AAV–GFP vs DF2‐CKO + AAV–*YTHDF2*: *p* > 0.999; F: one‐way ANOVA, *F*(3, 46) = 2.377, *p* = 0.082). G,H) Contextual (G) and auditory (H) fear memory assessed 24 h after fear training (*n* = 14, 13, 11, 12 mice, G: one‐way ANOVA, *F*(3, 46) = 10.49, *p* <0.0001, post hoc: Bonferroni's test, Control + AAV–GFP vs Control + AAV–*YTHDF2*, *p* = 0.043, DF2‐CKO + AAV–GFP vs DF2‐CKO + AAV–*YTHDF2*, *p* = 0.017, Control + AAV–GFP vs DF2‐CKO + AAV–GFP, *p* = 0.026, Control + AAV–GFP vs DF2‐CKO + AAV–*YTHDF2*: *p* > 0.999; H: one‐way ANOVA, *F*(3, 46) = 1.956, *p* = 0.134). I) Schematic diagram illustrating the experimental timeline of AAV–*YTHDF2* or AAV–GFP injection followed by fear conditioning and subsequent patch‐clamp recording. J) Representative traces of sEPSCs. K,L) Cumulative probability plots and bar graph (inside) indicated both decreased sEPSCs frequency (K) and amplitude (L) in hippocampal CA1 pyramidal neurons from DF2‐CKO mice hippocampus injected with AAV–*YTHDF2* after fear conditioning (*n*_DF2‐CKO+AAV–GFP_ = 21 cells, 4 mice, *n*_DF2‐CKO+AAV–_ *_YTHDF2_* = 23 cells, 4 mice; unpaired *t*‐test, *t*_42_ = 4.253, *p* < 0.001, KS test, *p* < 0.0001 for frequency; unpaired *t*‐test, *t*_42_ = 2.337, *p* = 0.024, KS test, *p* < 0.0001 for amplitude). M) Representative traces (left) and I–O curves (right) showed the amplitudes of evoked EPSC at CA3–CA1 pathway from DF2‐CKO mice hippocampus injected with AAV–GFP or AAV–*YTHDF2* after fear conditioning (*n*_DF2‐CKO+AAV–GFP_ = 15 cells, 2 mice, *n*_DF2‐CKO+AAV–_ *_YTHDF2_* = 13 cells, 2 mice; unpaired *t*‐test, *t*_8_ = 3.776, *p* = 0.005). N) Representative traces (left) and histogram (right) of paired‐pulse ratio (PPR) from DF2‐CKO mice hippocampus injected with AAV–GFP or AAV–*YTHDF2* after fear conditioning (*n*_DF2‐CKO+AAV–GFP_ = 15 cells, 2 mice, *n*_DF2‐CKO+AAV–_ *_YTHDF2_* = 14 cells, 2 mice; unpaired *t*‐test, *t*_27_ = 3.665, *p* = 0.001). O) Representative traces (left) and summary plots (right) showed LTP induced by HFS (1 train) at CA3–CA1 pathway in DF2‐CKO mice injected with AAV–GFP or AAV–*YTHDF2* after fear conditioning (*n*_Control_ = 10 cells from 4 mice, *n*_DF2‐CKO_ = 10 cells from 4 mice; unpaired two‐tailed *t*‐test, *t*_18_ = 18.91, *p* < 0.0001). Data are presented as mean ± standard error. **p < 0.01; ***p < 0.001; ****p < 0.0001.

To confirm that the observed improvement in spatial learning and memory was attributed to the loss of *YTHDF2* specifically in the hippocampus, we investigated whether reintroducing *YTHDF2* to the hippocampus of adult DF2‐CKO mice would be adequate to reverse the phenotypes. We utilized adeno‐associated virus (AAV) carrying either flag‐tagged *YTHDF2* (AAV–*YTHDF2*) or a control fluorescent protein GFP (AAV–GFP) and stereotactically injected them into the bilateral hippocampus (**Figure** **3**A). The viral infection successfully restored *YTHDF2* expression across major hippocampal subregions, including CA1–CA3 and DG (Figure 3B; Figure S7A, Supporting Information). The restored levels of *YTHDF2* proteins were ≈80% of control levels (Figure 3C). In the fear conditioning test, *YTHDF2* reexpression in DF2‐CKO mice almost normalized contextual memory to control levels both 1 and 24 h after training, with no effect on auditory fear memory (Figure 3D–H) or the motor activity and anxiety level in open field test (Figure S7B,C, Supporting Information) and light–dark box test (Figure S7D,E, Supporting Information). Interestingly, the overexpression of *YTHDF2* in the hippocampus of control mice resulted in impaired short‐term and long‐term contextual fear memory (Figure 3E,G), while not affecting the auditory fear memory (Figure 3F,H), consistent with the speculation that *YTHDF2* is a negative regulator of learning and memory in the hippocampus.

Additionally, we investigated the effects of *YTHDF2* reexpression on synaptic transmission in DF2‐CKO hippocampus. Whole‐cell patch‐clamp recordings revealed that reexpression of *YTHDF2* significantly reduced both the frequency and amplitude of sEPSCs after fear learning (Figure 3I–L) but left sIPSCs unaffected (Figure S7F–H, Supporting Information). Consistent with these findings, *YTHDF2* reexpression also diminished I–O curves and reversed PPR in CA1 pyramidal neurons (Figure 3M,N). To assess the functional consequences, we repeated LTP experiments in DF2‐CKO mice expressing either AAV–*YTHDF2* or AAV–GFP. Following a moderate training protocol (0.8 mA, 1 s, 3 pairs), HFS‐induced LTP—which was occluded in DF2‐CKO mice—was fully restored by *YTHDF2* reexpression (Figure 3O). Together, these results indicate that *YTHDF2* negatively regulates learning and memory by selectively suppressing excitatory synaptic transmission.

### 2.4 Loss of *YTHDF2* Enhances the Stability of Synaptic‐Function‐Related Target mRNAs

**Figure 4:**
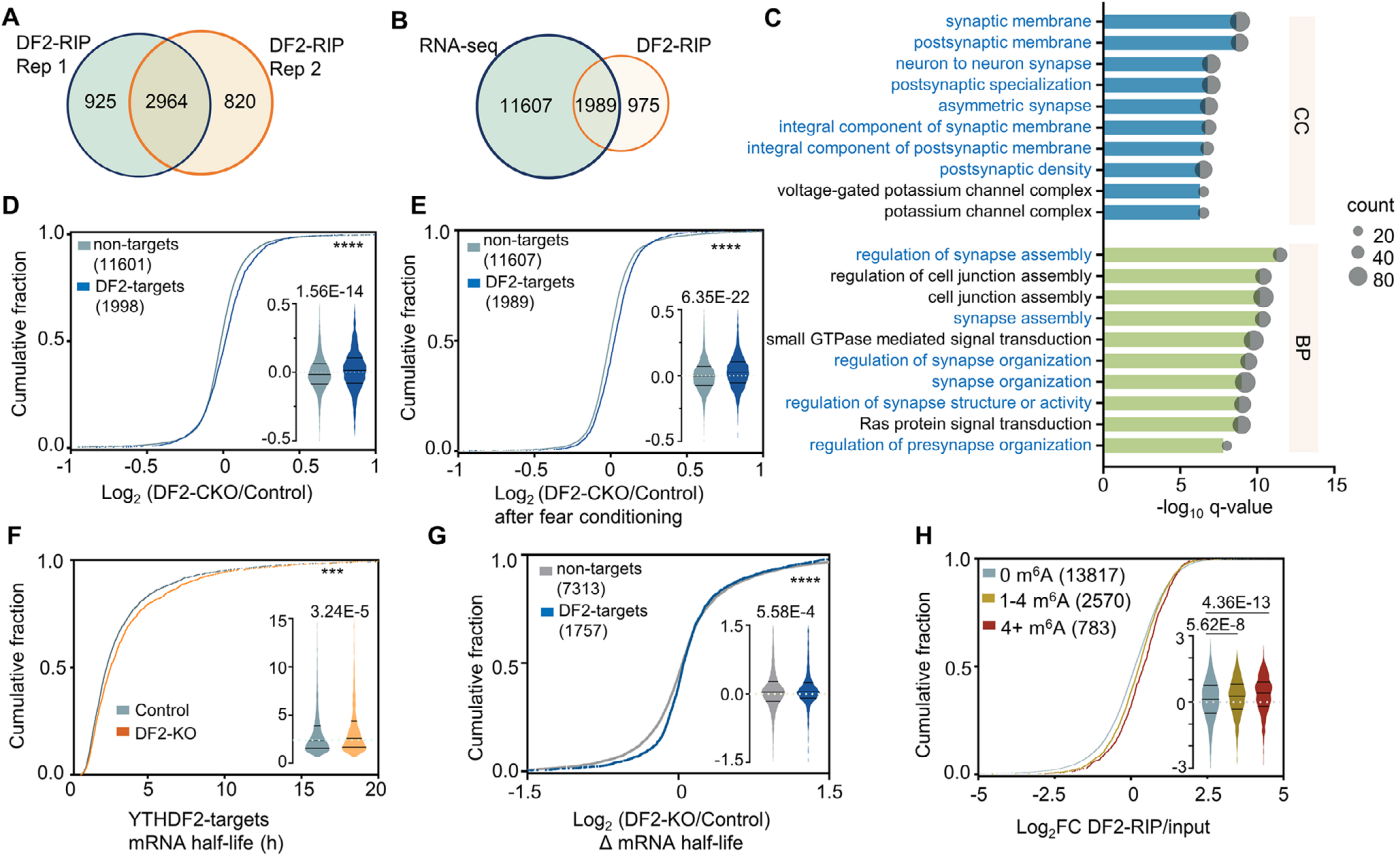
Loss of *YTHDF2* enhances the stability of synaptic function related target mRNAs. A) Venn plot showing the overlap of high confidence *YTHDF2*‐binding target mRNAs between replicate 1 and replicate 2 in *YTHDF2* RIP‐seq. Significant targets were defined as foldchange (IP/input) >1.8 and FDR < 0.05. B) Venn plot showing the overlap of total mRNAs from RNA seq after fear conditioning and *YTHDF2* RIP‐seq. mRNAs with FPKM ≥ 0.5 in RNA‐seq were included. C) GO enrichment analysis of overlapped genes identified from RNA seq and *YTHDF2* RIP‐seq in (B). Top ten enriched BP and CC terms were showed. D) Cumulative distribution and violin plot (inside) showing the distribution of log_2_‐fold change in nontarget mRNAs and DF2 RIP target mRNAs upon *YTHDF2* depletion (Mann–Whitney test, *U* = 1.034E7, *p* = 1.56E−14, KS test, *Z* = 4.767, *p* < 0.0001). E) Cumulative distribution and violin plot (inside) showing the distribution of log2‐fold change in nontarget mRNAs and DF2 RIP target mRNAs upon *YTHDF2* depletion after fear conditioning. (Mann–Whitney test, *U* = 9986652, *p* = 6.35E−22, KS test, *Z* = 4.808, *p* < 0.0001). F) Cumulative distribution and violin plot (inside) of mRNA half‐life of *YTHDF2* targets (1757) in hippocampal primary cultured neurons from either control or DF2‐CKO mice (Mann–Whitney test, *U* = 1418545, *p* = 3.24E−5, KS test, *Z* = 2.176, *p* = 1.54E−4). G) Cumulative distribution and violin plot (inside) showing the distribution of log_2_‐fold change of ΔmRNA half‐life between DF2‐CKO and control neurons for nontargets, and *YTHDF2* targets (Mann–Whitney test, *U* = 6084342, *p* = 5.58E−4, KS test, *Z* = 3.410, *p* = 1.59E−10). H) Cumulative distribution plot and violin plot (inside) of the enrichment in *YTHDF2* RIP over input for transcripts with 0 (gray), 1–4 (yellow), or 4+ (red) m^6^A‐CLIP‐seq peaks (Kruskal–Wallis test, *K* = 78.97, *p* = 7.11E−18, Dunn's test multiple comparison of two or samples, non vs low m^6^A: *p* = 5.62E−8, non vs high m^6^A: *p* = 4.36E−13). Data in violin plots are presented as the median and quartiles. ***p < 0.001; ****p < 0.0001.

*YTHDF2* has been identified as a m^6^A reader protein and is known to play a crucial role in promoting the degradation of m^6^A‐modified target mRNAs.^[^ ^12^, ^15^ ^]^ To elucidate its functional significance in learning and memory, we conducted RNA immunoprecipitation sequencing (RIP‐seq) to map target transcripts bound by *YTHDF2* and RNA‐sequencing (RNA‐seq) to assess their expression level in the hippocampus. The RIP‐seq results exhibited high reproducibility between two biological replicates (Figure S8A,B, Supporting Information), allowing for the confident identification of 2964 mRNAs as *YTHDF2* targets (**Figure** **4**A; Table S1, Supporting Information). Among these targets, 1989 were detected in RNA‐seq data following fear conditioning (Figure 4B; Table S2, Supporting Information). Gene ontology (GO) functional annotation of those *YTHDF2* targets revealed a significant enrichment in processes associated with synapse assembly, organization, and synaptic membrane (Figure 4C; Table S1, Supporting Information), consistent with the observed changes in memory and synaptic plasticity in DF2‐CKO mice. Cumulative and violin plots were employed to visualize the mRNA expression patterns in RNA‐seq. Notably, a significant increase in mRNA abundance for *YTHDF2* targets was observed in the *YTHDF2* knockdown sample compared to the nontarget mRNAs at the basal level (Figure 4D), and this increase was also evident after fear conditioning (Figure 4E). Additionally, among the significantly differentially expressed genes (DEGs) in RNA‐seq, most of the *YTHDF2* targets were upregulated in DF2‐CKO mice, while the proportion of upregulated DEGs was notably lower in nontargets (Figure S8C, Supporting Information).

Subsequently, we conducted RNA half‐life profiling by analyzing RNA‐seq data obtained from primary cultured hippocampus neurons at various time points following actinomycin D (ActD) treatment to inhibit transcription. Our analysis revealed a prolonged half‐life of *YTHDF2* targets in DF2‐CKO neurons compared to the control group (Figure 4F; Table S3, Supporting Information). Moreover, the half‐life of *YTHDF2* targets were significantly increased compared to nontarget mRNAs (Figure 4G; Table S3, Supporting Information). Among those *YTHDF2* targets with prolonged lifetime, the GO enrichment analysis also showed substantial enrichment in synapse assembly, organization, and synaptic membrane (Figure S8D and Table S3, Supporting Information). The prolonged half‐life of some synaptic‐function‐related genes (including *Bdnf*, *Grin2b*, *Slitrk1*, *Slitrk2*, and *Shank2*) shown in the GO analysis were further confirmed by reverse transcriptase quantitative polymerase chain reaction (RT‐qPCR) (Figure S8E–I, Supporting Information). Additionally, a positive correlation was observed between the number of m^6^A crosslinking and immunoprecipitation (CLIP)‐seq peaks (data from previous study^[^ ^11^ ^]^) and the RNA enrichment in the *YTHDF2* RIP sample relative to the input (Figure 4H; Table S1, Supporting Information), suggesting that a higher number of m^6^A modification peaks on mRNA is associated with increased abundance in the *YTHDF2* RIP‐seq. Collectively, these results provide compelling evidence for the pivotal role of *YTHDF2* in mediating the degradation of m^6^A‐modified mRNAs involved in synaptic functions. Therefore, depletion of *YTHDF2* results in enhanced stability of these target mRNAs, which may contribute to the enhanced learning and memory in DF2‐CKO mice.

### 2.5 Activity‐Dependent Protein Synthesis Was Enhanced in DF2‐CKO Mice Hippocampus

**Figure 5:**
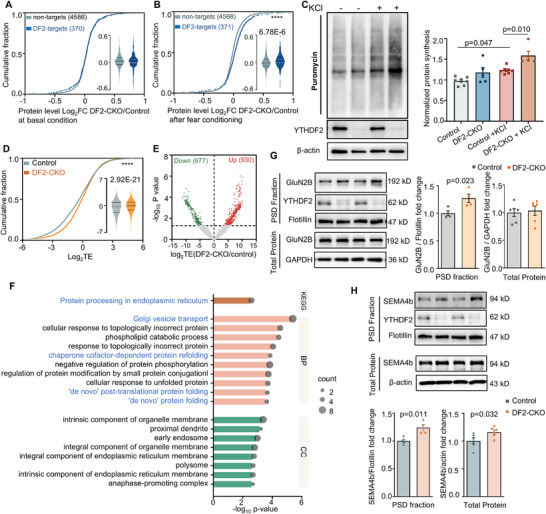
The activity‐dependent protein synthesis is enhanced in DF2‐CKO mice hippocampus. A) Cumulative distribution and violin plot (inside) showing the log_2_‐fold change in protein levels between DF2‐CKO and control mice in hippocampal tissue under basal conditions. Data are stratified by nontargets versus *YTHDF2* RIP targets (Mann–Whitney test, *U* = 840670, *p* = 0.770, KS test, *Z* = 0.741, *p* = 0.642). B) Cumulative distribution and violin plot (inside) showing the log_2_‐fold change in protein levels between DF2‐CKO and control mice in hippocampal tissue after fear conditioning. Data are stratified by nontargets versus *YTHDF2* RIP targets (Mann–Whitney test, *U* = 728491, *p* = 6.78E−6, KS test, *Z* = 2.495, ^****^ *p* = 7.87E−6). C) Representative blot (left) and quantification data (right) of puromycin signal in KCl‐treated primary cultured hippocampus neurons from either control or DF2‐CKO mice (*n* = 7, 5, 7, 5 wells, one‐way ANOVA, *F*(3, 20) = 12.64, *p* <0.0001, post hoc: Turkey's test, Control vs Control + KCl, *p* = 0.047, Control + KCl vs DF2‐CKO + KCl, *p* = 0.010). D) Cumulative distribution and violin plot (inside) showing the log_2_‐translational efficiency (TE) of genes in control and DF2‐CKO mice hippocampus at 1 h after fear conditioning (Mann–Whitney test, *U* = 2.37E7, *p* = 2.92E−21, KS test, *Z* = 6.232, *p* <0.0001). E) Volcano plot showing the significantly changed TE in DF2‐CKO mice compared to control. Significantly changed TE was defined as log_2_TE > 1 and *p* < 0.05. F) KEGG and GO enrichment analysis of upregulated proteins identified from hippocampus proteomics at 4 h after fear conditioning. Top ten enriched BP and CC terms were showed. Upregulated proteins were defined as fold change (DF‐CKO/Control) > 1.2 and *p* <0.05. G,H) Representative western blot (left) and quantification data (right) of GluN2B (G) and *SEMA4B* (H) expression in PSD fraction and total hippocampal lysates from control and DF2‐CKO mice at 4 h after fear conditioning (G: PSD fraction: *n* = 4 mice per group, unpaired two‐tailed *t*‐test, *t*_6_ = 3.036, *p* = 0.023; total protein: *n* = 6 mice per group, unpaired two‐tailed *t*‐test, *t*_10_ = 0.329, *p* = 0.749; H: PSD fraction: *n* = 4 mice per group, unpaired two‐tailed *t*‐test, *t*_6_ = 3.600, *p* = 0.011; total protein: *n* = 6 mice per group, unpaired two‐tailed *t*‐test, *t*_10_ = 2.492, *p* = 0.032). Data in violin plots are presented as the median and quartiles. Data in bar plots are presented as mean ± standard error. ****p < 0.0001.

To further investigate whether *YTHDF2*‐deletion‐induced stabilization of target mRNAs leads to protein expression changes, we conducted proteomic analysis in hippocampal tissues from DF2‐CKO and control mice both at the basal level and following fear conditioning training. Interestingly, at the basal condition, there was no obvious difference in the abundance of *YTHDF2* targets at the protein level, but their abundance significantly increased after fear conditioning in DF2‐CKO mice (**Figure** **5**A,B; Table S4, Supporting Information), suggesting a potential dynamic regulation of protein synthesis in response to learning stimulation. To gain further insights into the translation regulation after *YTHDF2* knockout, we performed in vitro surface sensing of translation (SUnSET)^[^ ^27^ ^]^ assay in cultured primary hippocampal neurons to measure stimulus induced protein synthesis. This approach also allowed us to eliminate potential confounding effects from nonneuronal cells, such as astrocytes, present in hippocampal tissue. We detected significantly higher puromycin incorporation in KCl‐treated DF2‐CKO neurons compared to control neurons, but not under basal conditions (Figure 5C), indicating that enhanced protein synthesis in DF2‐CKO neurons depends on depolarization. Ribosome profiling of the hippocampus after fear conditioning further revealed increased translational efficiency (TE) in DF2‐CKO mice (Figure 5D; Table S5, Supporting Information). Notably, transcripts with significantly upregulated TE (930, 58%) substantially outnumbered those with downregulated TE (677, 42%) (Figure 5E), demonstrating stronger translation enhancement in DF2‐CKO mice following learning stimuli. Although *YTHDF2* has been primarily implicated in mRNA degradation rather than translation regulation,^[^ ^12^, ^28^, ^29^ ^]^ we propose that the observed global translation increase in DF2‐CKO mice may stem from elevated mRNA abundance and require learning associated stimuli.

Proteomic analysis in hippocampal tissues after fear conditioning identified 191 differentially expressed proteins (DEPs), with 100 upregulated and 91 downregulated. The relatively small number of DEPs detected in our hippocampal tissue proteomic analysis may reflect the confounding influence of nonneuronal cells (e.g., astrocytes) in whole‐tissue samples. Enrichment analysis revealed that downregulated proteins were strongly associated with mRNA metabolic, destabilization, and stress granule/P‐body functions (Figure S9A, Supporting Information). Accordingly, hippocampal levels of P‐body marker decapping mRNA 1A (DCP1a) and stress granule marker T‐cell‐restricted intracellular antigen‐1 (TIA1) were both reduced in DF2‐CKO mice (Figure S9B, Supporting Information), suggesting *YTHDF2* deletion coordinately suppresses mRNA turnover pathways. Conversely, upregulated proteins were enriched in protein processing, Golgi vesicle transport, and protein folding (Figure 5F; Table S4, Supporting Information), aligning with the observed enhancement in protein production after fear conditioning.

Given that local protein synthesis plays a key role in consolidating neuronal activity into synaptic structure and functional changes, and thousands of m^6^A‐modified mRNAs have been identified to localize to synaptic compartments,^[^ ^30^, ^31^ ^]^ including half of *YTHDF2* targets (Figure S9D, Supporting Information), we next investigated whether this stimulus‐induced protein synthesis would be observed in the synaptic compartments. Two synaptic‐function closely related *YTHDF2* targets, *Grin2B* and *SEMA4B*, were selected for characterization. By isolating the hippocampal postsynaptic density (PSD) fraction at 4 h after fear conditioning, we observed an increased level of GluN2B in the PSD fraction but with no obvious alterations in the total protein level (Figure 5G). Additionally, *SEMA4B* was increased in both PSD fraction and total protein level (Figure 5H), consistent with the findings in proteomics (Figure S9C, Supporting Information). Collectively, these results suggest that learning‐stimulus‐induced protein synthesis was more evident at the synaptic compartments in DF2‐CKO mice hippocampus.

### 2.6 Knockdown of *SEMA4B*, a Downstream Target of *YTHDF2*, Reverses the Enhanced Memory Formation Observed in DF2‐CKO Mice

**Figure 6:**
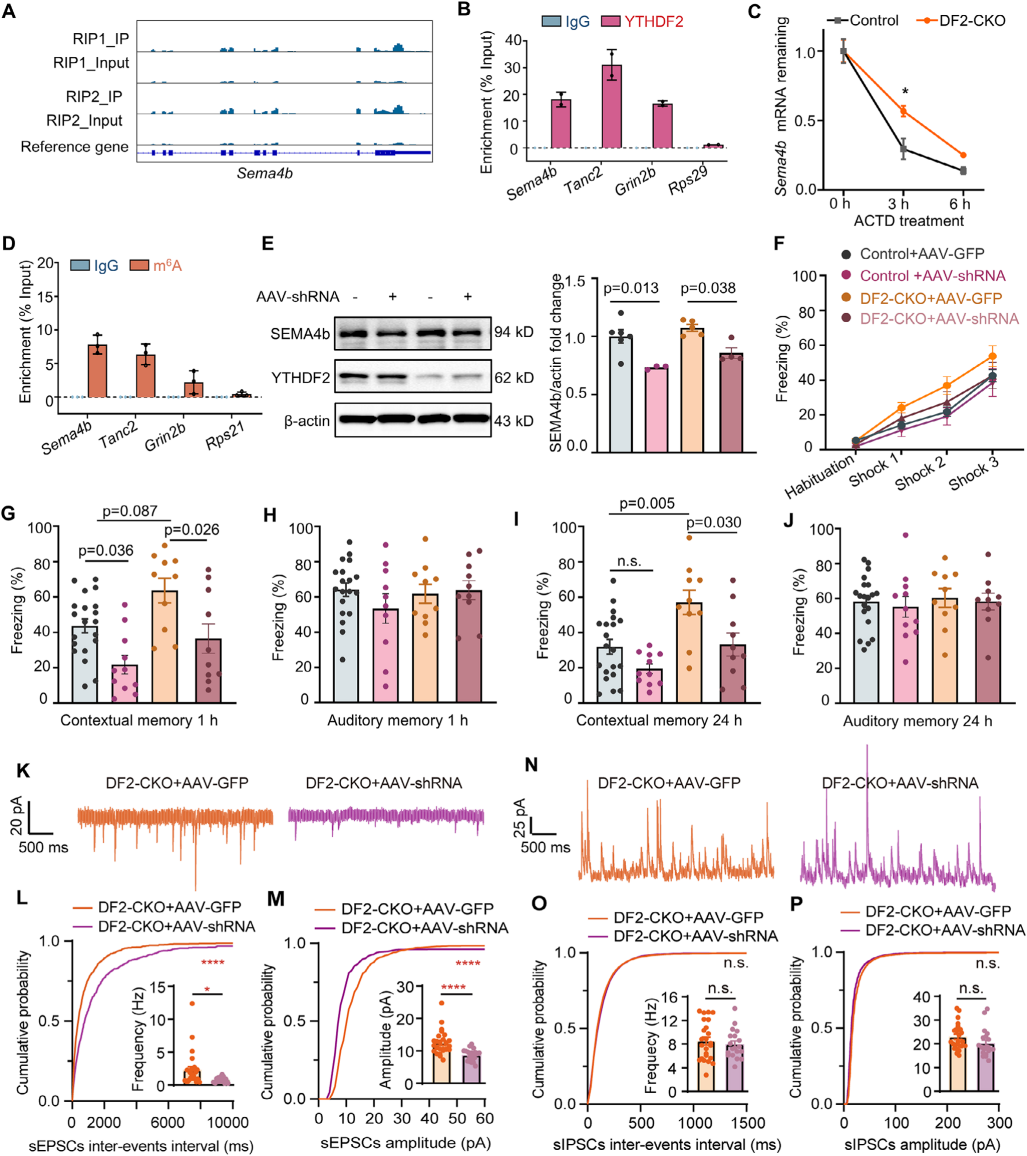
Knockdown of *SEMA4B* reverses the phenotype of DF2‐CKO mice. A) IGV visualization of *YTHDF2* binding peaks on *SEMA4B* mRNA in *YTHDF2* RIP‐seq. The binding peaks were enriched in IP samples compared to input. RIP1 and RIP2 represented two biological replicates of RIP‐seq. B) RIP‐qPCR analysis shows significant enrichment of *SEMA4B*, *Tanc2*, and *Grin2b* in *YTHDF2*‐immunoprecipitated samples compared to the negative control *RPS29* (not identified as a *YTHDF2* target in RIP‐seq), indicating specific binding of *YTHDF2* to these transcripts. C) The mRNA level of *SEMA4B* was analyzed by qPCR in Actinomycin‐D (ActD)‐treated primary neurons from control and DF2‐CKO mice (*n* = 3 cell dishes per group, repeated two‐way ANOVA, group factor: *F*(1, 4) = 11.14, *p* = 0.029, post hoc: Bonferroni's test, 3 h: *p* = 0.024). D) MeRIP‐qPCR analysis shows significant enrichment of *SEMA4B*, *TANC2*, and *Grin2b* in m^6^A‐immunoprecipitated samples compared to the negative control *Rps21* (not identified as a m^6^A‐modified target in meRIP‐seq), indicating the presence of m^6^A modification on these transcripts. E) Representative blot (left) and quantification data (right) of *SEMA4B* expression in hippocampus of mice injected with AAV–*SEMA4B* shRNA (*n* = 6, 3, 5, 4 mice, one‐way ANOVA, *F*(3, 14) = 8.778, *p* = 0.002, Bonferroni's test: Control + AAV–GFP vs Control + *SEMA4B* shRNA, *p* = 0.013, DF2‐CKO + AAV–GFP vs DF2‐CKO + AAV–*SEMA4B* shRNA, *p* = 0.038). F) The freezing curves of DF2‐CKO mice injected with AAV–*SEMA4B* shRNA during fear conditioning (*n* = 20, 11, 10, 10 mice, repeated two‐way ANOVA, group: *F*(3, 47) = 2.558, *p* = 0.066). G,H) Contextual (G) and auditory (H) fear memory assessed 1 h after fear training (G: *n* = 20, 11, 10, 10 mice, one‐way ANOVA, *F*(3, 47) = 7.72, *p* < 0.001, Bonferroni's test: Control + AAV–GFP vs Control + AAV–*SEMA4B* ShRNA, *p* = 0.036, Control + AAV–GFP vs DF2‐CKO + AAV–GFP, *p* = 0.087, DF2‐CKO + AAV–GFP vs DF2‐CKO + AAV–*SEMA4B* ShRNA, *p* = 0.036; H: *n* = 20, 11, 10, 10 mice, one‐way ANOVA, *F*(3, 45) = 0.7619, *p* = 0.521). I,J) Contextual (I) and auditory (J) fear memory assessed 24 h after fear training (I: *n* = 20, 11, 10, 10 mice, one‐way ANOVA, *F*(3, 47) = 7.838, *p* < 0.001, Bonferroni's test: Control + AAV–GFP vs Control + AAV–*SEMA4B* shRNA, *p* = 0.442, Control + AAV–GFP vs DF2‐CKO + AAV–GFP, *p* = 0.005, DF2‐CKO + AAV–GFP vs DF2‐CKO + AAV–*SEMA4B* shRNA, *p* = 0.030; J: *n* = 20, 11, 10, 10 mice, one‐way ANOVA, *F*(3, 47) = 0.1705, *p* = 0.916). K) Representative traces of sEPSCs. L,M) Cumulative probability plots and bar graph (inside) indicated both decreased sEPSCs frequency (L) and amplitude (M) in CA1 pyramidal neurons from DF2‐CKO mice with AAV–*SEMA4B* expression (*n*_DF2‐CKO+AAV–GFP_ = 26 cells, 3 mice, *n*_DF2‐CKO+AAV–shRNA_ = 21 cells, 3 mice; unpaired *t*‐test, *t*_45_ = 2.652, *p* = 0.011, KS test, *p* < 0.0001 for frequency; unpaired *t*‐test, *t*_45_ = 4.287, *p* <0.0001, KS test, *p* < 0.0001 for amplitude). N) Representative traces of sIPSCs. O,P) Cumulative probability plots and bar graph (inside) showing unaffected inhibitory neurotransmission (*n*_DF2‐CKO+AAV–GFP_ = 24 cells, 3 mice, *n*_DF2‐CKO+AAV–shRNA_ = 19 cells, 3 mice; unpaired *t*‐test, *t*_41_ = 0.623, *p* = 0.537 for frequency; unpaired *t*‐test, *t*_41_ = 1.505, *p* = 0.140 for amplitude). Data are presented as mean ± standard error. *p < 0.001; ****p < 0.0001.

Given that the enhanced contextual memory and excitatory synaptic transmission in DF2‐CKO mice resulted primarily from increased transcripts abundance and translation of multiple *YTHDF2* targets—particularly those involved in synaptic function—we next investigated whether knocking down these targets could reverse the phenotype. We focused on synaptic‐related *YTHDF2* targets exhibiting prolonged mRNA half‐life or elevated protein levels in DF2‐CKO mice. Strikingly, several of these genes (*GRIN2B*,^[^ ^32^ ^]^ *SLITRK2*,^[^ ^33^ ^]^ *BDNF*,^[^ ^34^ ^]^ and *TANC2* ^[^ ^35^ ^]^) have established roles in learning and memory regulation, with hippocampal knockout known to impair memory function. Among these, *SEMA4B*, a member of the class 4 Semaphorin family, emerged as particularly interesting (Figure S9C, Supporting Information). Although *SEMA4B* interacts with PSD‐95^[^ ^36^ ^]^ and regulates glutamatergic synapses development,^[^ ^37^ ^]^ its direct role in learning and memory remains unexplored. *SEMA4B* mRNA was enriched in *YTHDF2* RIP sample (**Figure** **6**A,B), and its decay was significantly delayed following *YTHDF2* knockout (Figure 6C). MeRIP‐qPCR analysis shows significant enrichment of *SEMA4B* in m^6^A‐immunoprecipitated samples (Figure 6D), consistent with a recent absolute m^6^A quantification study detecting nine distinct m^6^A sites on *SEMA4B* specifically within synaptic regions.^[^ ^38^ ^]^ Based on these findings, we hypothesized that disrupting expression of *SEMA4B*, a strong *YTHDF2* target candidate, would partially reverse the enhanced learning and memory phenotype in DF2‐CKO mice. To test the impact of *SEMA4B* knockdown, we designed a shRNA expressing AAV vector targeting *SEMA4B* (AAV–shRNA–*SEMA4B*) and bilaterally injected it into the DF2‐CKO mice hippocampus (Figure S10, Supporting Information). Western blot confirmed efficient *SEMA4B* knockdown, with expression reduced by ≈30% (Figure 6E). Strikingly, *SEMA4B* knockdown impaired contextual fear memory in control mice (Figure 6G,I), demonstrating its direct role in memory formation. In DF2‐CKO mice, *SEMA4B* knockdown effectively reversed the enhanced contextual memory at both 1 and 24 h post‐training (Figure 6G–J), whereas AAV–Control‐injected DF2‐CKO mice retained elevated fear memory. To determine whether *SEMA4B* knockdown also rescued the synaptic effects of *YTHDF2* deletion, we performed whole‐cell patch‐clamp recordings from CA1 pyramidal neurons in DF2‐CKO mice injected with either AAV–shRNA–*SEMA4B* or AAV–GFP. *SEMA4B* knockdown significantly reduced both the frequency and amplitude of sEPSCs (Figure 6K–M), while leaving inhibitory synaptic transmission unaffected (Figure 6N–P). This aligns with prior findings that *SEMA4B* RNAi decreases the frequency and amplitude of mEPSCs.^[^ ^37^ ^]^

In summary, our profiling of *YTHDF2* targets in DF2‐CKO mice revealed *SEMA4B* as a previously unrecognized regulator of learning and memory. Downregulating synaptic *YTHDF2* targets—particularly *SEMA4B*—was sufficient to normalize the enhanced memory and excitatory synaptic transmission in DF2‐CKO mice. These results establish a mechanism whereby *YTHDF2* regulates memory by controlling mRNA stability of key synaptic plasticity‐related genes.

## 3 Discussion

Our study revealed a novel role of *YTHDF2* as a negative regulator of hippocampus‐dependent learning and memory. We provided several lines of evidence to support this notion. First, conditional knockout of *YTHDF2* in the forebrain enhanced spatial learning and memory, and increased excitatory synaptic transmission in hippocampal CA1 pyramidal neurons. Second, selectively reexpression of *YTHDF2* in CKO mice hippocampus suppressed spatial memory performance and excitatory synaptic transmission. Third, loss of *YTHDF2* function was sufficient to increase synaptic‐function‐related mRNA stability and activity‐induced local protein synthesis. Finally, knockdown *YTHDF2* downstream target *SEMA4B*, reversed the phenotype of *YTHDF2*‐CKO mice.

In a recent study, a distinct role for *YTHDF2* in learning and memory within the hippocampal DG was reported. The study found that DG‐specific deletion of *YTHDF2* impaired memory formation by regulating the elongation of mossy fibers and axons of DG granule cells. By contrast, CA3‐ and CA1‐specific knockout of *YTHDF2* did not induce significant alterations in spatial memory in mice.^[^ ^19^ ^]^ The differential effects observed in subregion‐specific knockout compared to global knockout of *YTHDF2* in the hippocampus may be attributed to distinct regulatory mechanisms in different hippocampal subregions. This hypothesis is supported by the fact that subregion knockout of *YTHDF1* showed unchanged spatial memory,^[^ ^19^ ^]^ while global *YTHDF1* deletion impaired spatial memory.^[^ ^11^, ^12^, ^13^, ^14^, ^15^, ^16^, ^17^, ^18^, ^19^, ^20^ ^]^ Besides, different Cre lines exhibit varying recombination windows, which may influence phenotypic outcomes. Additionally, in contrast to *YTHDF2* knockout leading to enhanced fear memory, overexpression of *YTHDF2* in wild‐type mice hippocampus impaired fear memory formation (Figure 3E,G), further supporting the idea that *YTHDF2* acts as a negative regulator of hippocampus‐dependent learning and memory. Finally, consistent with the observation that *YTHDF2* knockout in hippocampus leads to increased spine density and synaptic transmission of hippocampal neurons, deletion of *YTHDF2* increased axon growth in cerebellar granule cell (CGC) and dendrite branching and synapse number in retina ganglion cells, leading to improved motor coordination ability and visual function in mice, respectively.^[^ ^39^ ^]^

Though global knockout of *YTHDF2* results in embryonic lethality, and *YTHDF2* deletion in the embryonic neocortex may lead to impaired NSPC proliferation and differentiation potential.^[^ ^16^ ^]^ Our results demonstrate that conditional deletion of *YTHDF2* in the forebrain does not affect the proliferation of DG neurons during early postnatal hippocampal development (Figure S2C,D, Supporting Information). This aligns with a recent study,^[^ ^40^ ^]^ which found that *YTHDF2* knockout in proliferating DG NSCs leading to a modest increase in the adult NSC pool in the DG without altering NSC proliferation at P30, and also no change in the neuronal subtype fate specification.

*YTHDF2* has been reported to colocalize with cytoplasmic P‐bodies and stress granules,^[^ ^12^, ^14^ ^]^ both of which serve as crucial hubs for mRNA degradation and translational regulation. These structures house decapping factors, translational repressors, and components of the 5′–3′ mRNA decay machineries.^[^ ^41^, ^42^ ^]^ Consistently, in the present study, we observed that *YTHDF2* knockout in hippocampal neurons led to increased abundance and extended half‐life of target mRNAs, which were enriched in synaptic‐related functional pathways. Moreover, the levels of P‐body marker DCP1a and stress granule marker TIA1 were both reduced in the hippocampus of DF2‐CKO mice (Figure S9B, Supporting Information), suggesting *YTHDF2* depletion may alter the P‐bodies and stress granules, leading to a dynamic regulation of mRNA metabolism and translation involving P‐bodies and stress granules.^[^ ^42^ ^]^ Interestingly, a similar decrease in DCP1a has been observed in *YTHDF2* knockdown goat embryos.^[^ ^43^ ^]^ However, depletion of all three *YTHDF*s (*YTHDF1*, *YTHDF2*, and *YTHDF3*) led to an increase in DCP1a levels^[^ ^29^ ^]^ and enhanced mRNA stability,^[^ ^29^, ^44^ ^]^ indicating that the functions of these three *YTHDF*s in mediating mRNA degradation and their relationship with P‐bodies are complex and require further investigation.

The local distribution and translation of mRNAs at the dendrites and synapse plays a crucial role in synaptic processes and memory formation.^[^ ^45^ ^]^ Recently, mRNA stability and m^6^A have been reported as major determinants of subcellular mRNA localization in neurons,^[^ ^46^ ^]^ and many of the m^6^A regulatory proteins, including *METTL14*, *FTO*, *YTHDF1*, *YTHDF2*, and *YTHDF3*, along with m^6^A‐methylated transcripts, are localized in dendritic spines.^[^ ^47^ ^]^ Thus, the contribution of m^6^A to local translation in neurons is unsurprising.^[^ ^48^ ^]^ Notably, both *FTO* and *YTHDF1* have been previously demonstrated to influence local translation in axons and synapses.^[^ ^11^, ^30^, ^31^ ^]^ *YTHDF1* and *YTHDF2* have been found to work synergistically through regulating local translation in CGC axons to control cerebellar parallel fiber development.^[^ ^30^ ^]^ Our present results underscore that activity‐induced protein synthesis in DF2‐CKO mice occurs both at the whole cell level and at the synaptic regions, providing new evidence for m^6^A‐ and *YTHDF2*‐dependent regulation of local translation. While the prevailing view suggests that *YTHDF2* does not directly influence mRNA translation.^[^ ^44^ ^]^ A recent study reported a RNA‐stability‐independent role for *YTHDF2* in promoting protein translation in ovarian cancer cells without altering mRNA abundance.^[^ ^49^ ^]^ By contrast, our study observed a significant increase in target mRNA abundance and stability, suggesting that indirect mechanisms downstream of m^6^A‐mediated mRNA decay may contribute to translational changes.^[^ ^50^ ^]^ Given *YTHDF1*’s established role in activity‐dependent translation regulation, future studies should investigate whether *YTHDF1* participates in this process.

*SEMA4B*, a member of the class 4 Semaphorin family, has been shown to regulate glutamatergic synapse development, but its direct role in learning and memory remained unexplored. Through rigorous validation including *YTHDF2* RIP‐qPCR, meRIP‐qPCR, RNA stability assays, and Western blotting, we confirmed *YTHDF2*’s binding and regulatory control of *SEMA4B*. Our functional studies demonstrated that *SEMA4B* knockdown impaired contextual fear memory in both control and DF2‐CKO mice (Figure 6G–I), establishing its direct role in memory formation. Electrophysiological analysis further revealed *SEMA4B* knockdown significantly reduced both the frequency and amplitude of sEPSC in CA1 pyramidal neurons (Figure 6K–M), while leaving inhibitory transmission unchanged (Figure 6N–P). These findings not only demonstrate *SEMA4B*’s essential role in memory formation but also align with previous reports showing that *SEMA4B* RNAi decreases mEPSC frequency and amplitude,^[^ ^37^ ^]^ indicating *SEMA4B* could regulate both presynaptic and postsynaptic function. Our study provides the first direct evidence linking *SEMA4B* to learning and memory processes through *YTHDF2*‐mediated regulation.

Under basal condition, the bulk proteomic analysis revealed no significant change in hippocampal levels of *YTHDF2* target proteins (Figure 5A). However, we observed enhanced sEPSC frequency (Figure 2B) and increased presynaptic transmitter release (Figure S6E, Supporting Information) in DF2‐CKO mice. Consistent with these findings demonstrating synaptic strengthening, dendritic spine density was also increased (Figure 2D), suggesting a greater number of excitatory synapses. Following learning stimuli, DF2‐CKO mice showed amplified: dendritic spine density, excitatory synaptic transmission, abundance of *YTHDF2* target mRNAs, translation activity and protein expression of *YTHDF2* targets, spatial memory performance. This apparent discrepancy can be explained by several factors. 1) Stimulus threshold effect—basal environment stimuli are insufficient to fully activate translational machinery, while learning stimuli unlock full translational potential, driving significant increases in synaptic protein expression and functional outcomes. 2) Cellular heterogeneity—nonneuronal cells (e.g., astrocytes with low *YTHDF2* expression), comprise >50% of hippocampal cells and may mask neuron‐specific translational changes in whole‐tissue proteomics. 3) Local protein synthesis—synapse‐localized translation during development may drive enhanced synaptic transmission and spine density increases that escape detection in bulk‐tissue analyses. 4) Downstream target involvement—although *YTHDF2*’s target *SEMA4B* interacts with PSD‐95 and is primarily postsynaptic, its knockdown significantly reduced both the frequency and amplitude of sEPSCs, suggests *SEMA4B* regulates both presynaptic and postsynaptic function. Furthermore, additional *YTHDF2* targets may have similar effects, which requires systematic investigation in future studies.

In conclusion, the current study revealed that *YTHDF2* deletion in the hippocampus led to increased stability of target mRNAs, thereby contributing to the enhanced protein synthesis elicited by learning stimulus at the synapse. This ultimately resulted in improved spatial memory and excitatory synaptic transmission. Our findings shed light on the specific m^6^A regulation mediated by *YTHDF2*, which underlies synaptic plasticity and memory formation. This offers new insights into the molecular basis of learning and cognition, and provides a basis for developing novel interventions for memory‐related cognitive disorders.

## 4 Experimental Section

### Generation of *YTHDF2*‐CKO Mice and Genotyping

The *YTHDF2^flox/flox^* mice with C57BL/6J genomic background, previously described,^[^ ^23^ ^]^ were generated in Dr. Bin Shen's Laboratory. *Emx1‐Cre* mice (The Jackson Laboratory stock 005628, generously provided by Dr. Longzhen Cheng at the Southern University of Science and Technology, Shenzhen, China) were bred and maintained on C57BL/6J genomic background. *YTHDF2^flox/flox^* mice were crossed with *Emx1‐Cre* mice to generate *YTHDF2^flox flox^Emx1‐Cre* mice. *YTHDF2^flox/ flox^Emx1‐Cre* and *YTHDF2^flox/flox^* breeding pairs produced offspring with *YTHDF2^flox/ flox^Emx1‐Cre* and *YTHDF2^flox/flox^* genotypes in a Mendelian ratio of 1:1. Mice carrying the *YTHDF2^flox/ flox^Emx1‐Cre* genotype were referred to as DF2‐CKO mice, which exhibited specific deletion of the conditional *YTHDF2* alleles in the developing forebrain, including both the cortical and hippocampal regions. Littermates carrying the *YTHDF2^flox/flox^* genotype were used as controls for comparison.

For genotyping, mice were weaned at third postnatal week and genotyped by PCR. *Emx1‐Cre* and wild‐type alleles were detected by PCR assays according to the Jackson Laboratory genotyping protocol with primers oIMR1084 and oIMR1085 for mutant and oIMR4170 and oIMR4171 for wild type, amplified a 378 bp fragment (wild‐type) and a 102 bp fragment for mutant. *YTHDF2^flox/flox^* and wild‐type alleles were detected with primers *YTHDF2* 1F and *YTHDF2* 1R, amplified a 612 bp fragment (wild‐type) and a 772 bp fragment for *flox* mutant.

All animal procedures were conducted in accordance with animal care guidelines approved by the Institutional Animal Care and Use Committee of Shenzhen Institute of Advanced Technology, Chinese Academy of Sciences. The animals were maintained under a regular 12 h light–dark cycle with lights on at 08:00, at a temperature of 21 ± 1 °C, and in an environment with 50 ± 5% humidity. Animals were provided ad libitum access to water and food.

### Fear Conditioning

Fear conditioning was carried out according to a previous study with some modifications.^[^ ^51^, ^52^ ^]^ All experiments were carried out in two contexts: context A and context B. The conditioning chamber (context A, 26 × 26 × 40 cm^3^) was sound‐attenuating and electrical foot shock was delivered via the stainless grid floor. The test chamber (context B, 26 × 26 × 40 cm^3^) was brightly lit and consisted of a flat white plastic floor. The chambers were cleaned with 70% ethanol before each session. Mice were first handled for 3 min each day for three consecutive days before behavior tests. On the day of training, mice were placed into context A and allowed to freely explore for 3 min (habituation), followed by fear training process, specifically, a tone CS (2800 Hz, 75 dB) was presented for 29 s that coterminated with a single electric foot shock (US, 0.8 mA, 1 s). This conditioning trial was repeated for 1/3/5 times (indicated in each figure legend) and the interval between CS–US was 30 s. Mice were returned to their home cage at 30 s after the last foot shock. Cued fear memory test was performed at 1 h for short‐term memory and 24 h for long‐term memory after fear training. Mice were placed into context A for 3 min to test contextual fear memory and then into context B for 3 min habituation, followed by the test sessions that began with the presentation of three tones (30 s each) with an interval of 30 s to test auditory memory. Fear memory was evaluated by measuring time spent in freezing during the test periods using the JLBehv‐FCSM‐4 (JiLiang).

### Barnes Maze

A Barnes maze was used to assess the spatial memory of mice. The maze had a white circular platform with 48 holes, each having visual cues. A removable escape box was placed under a specific hole. A camera tracked the mice during experiments. Before training, mice were familiarized with handling and maze escape. Training sessions involved placing mice in a central cylinder for 10 s and letting them explore the maze for 180 s. If a mouse found the escape box or explored for over 180 s, training stopped. Mice in the box stayed for an additional 60 s. If a mouse did not find the box, it was guided to the box and stayed for 180 s. The time taken to enter the escape box (latency) was recorded. Training occurred twice a day with a 2 h interval, lasting 6 days. After a 3 days break, a test trial was conducted without the escape box. Mice explored for 90 s, and the latency to explore the target hole and time spent in quadrants were recorded using ANY‐maze software. After each trial, mice rested in their home cage, and the maze and box were cleaned with a 70% ethanol solution to remove odor cues. Data collected included latency to enter the escape box and time spent in each quadrant during the test trial.

### Immunohistochemistry

For immunohistochemistry (IHC) of brain slices, mice were perfused with cold phosphate‐buffered saline (PBS) first and 4% paraformaldehyde (PFA) in PBS subsequently. The brain was removed and fixed in 4% PFA overnight at 4 °C and immersed in 30% sucrose in PBS for 1 day and repeated twice. The dehydrated tissue was frozen and stored at −80 °C. Brain tissue was sliced at 35 µm using freezing microtome (CM1950, Leica). The IHC procedures were performed according to the previous study^[^ ^52^ ^]^ with mild modification. Briefly, the slices were incubated with 0.3% Triton X‐100 in PBS for 15 min and then rinsed with PBS 3 times. Then, the tissues were incubated with blocking reagent (PBS, 0.3% Triton X‐100, 5% bovine serum albumin) for 2 h at room temperature, primary antibody solution overnight at 4 °C in dark place, and secondary antibody solution for 2 h at room temperature, the slices were mounted with histology mounting medium (Fluoroshield with DAPI, Sigma‐Aldrich, F6057). Imaging data were acquired by Zeiss LSM900‐Airyscan confocal fluorescent microscope (Zeiss, Germany) or Olympus VS120 whole slide scanner microscope (Olympus, Japan). Primary antibodies and secondary antibodies were diluted in antibody solution (0.3% Triton X‐100 and 1% bovine serum albumin in PBS). Primary antibodies: anti‐GFAP (Sigma‐Aldrich, G3893, 1:600), anti‐NeuN (Merck Millipore, MAB377 1:500), anti‐*YTHDF2* (Proteintech, 24744‐1‐AP‐100, 1:500), anti‐SATB2 (Abcam, ab92446, 1:500), anti‐CTIP2 (Abcam, ab18465, 1:750), anti‐Ki67 (Abcam, ab16667, 1:200), anti‐Brdu (Abcam, ab6326, 1:1000). Secondary antibodies: Alexa Fluor 594 (Goat anti‐Rabbit, Invitrogen, A‐11012), Alexa Fluor 488 (Goat anti‐Rat, Invitrogen, A11006, 1:500; Goat Anti‐Mouse, APExBIO, K1204), Alexa Fluor 647 (Goat anti‐Rabbit, Invitrogen, A‐21244).

### Western Blot

The procedures were processed according to our previous protocol with minor modifications.^[^ ^52^ ^]^ In brief, the mice were euthanized, and the brains were rapidly removed, and hippocampus tissue from each mice was homogenized with ice‐cold RIPA buffer (MCE, HY‐K1001) using a Tissue‐Tearor (BioSpec Products, 985370‐04). Samples were centrifuged at 12 000 *g* for 20 min, and the protein concentrations were examined by BCA Protein Assay Kit (Thermo Fisher, 23227). Then, 20 µg of protein samples were separated by sodium dodecyl sulfate (SDS)–polyacrylamide gel (10% or 15%), and then transferred to nitrocellulose membranes. After blocking with 5% bovine serum albumin (BSA) in Tris‐buffered saline containing 0.1% Tween‐20 (TBST) for 1 h at room temperature, the transferred membranes were incubated overnight at 4 °C with primary antibodies: anti‐*YTHDF2* (1:1000, Aviva Systems Biology, ARP67917_P050), anti‐*SEMA4B* (1:1000, Cell Signaling Technology, 13771S), anti‐DCP1a (1:1000, Proteintech, 22373‐1‐AP‐50), anti‐Tia1 (1:1000, PTM BIO, PTM‐5518), anti‐β‐actin (1:5000, Sigma, A5441), GAPDH (1:5000, Thermo Fisher Scientific, AM4300), GluN2B (1:1000, Cell Signaling Technology, 4207S), Flotillin 1 (Proteintech, 15571‐1‐AP). Following three washes with TBST, the membranes were then incubated with horseradish‐peroxidase (HRP)‐conjugated secondary antibodies (Anti‐mouse IgG HRP‐linked Antibody: CST, 7076s; Anti‐rabbit IgG HRP‐linked Antibody: CST, 7074s) in TBST with 3% BSA for 1 h at room temperature. After repeated washes, membranes were reacted with Western lightning Plus ECL (PerkinElmer, NEL104001EA). Images were scanned and captured with Micro Chemi (Bio‐rad, ChemiDoc XRS+) and the optical densities of detected bands were quantified using ImageJ software.

### Slice Preparation

Mice were anesthetized with 1% pentobarbital NEMBUTAL (100 mg kg^−1^) and then perfused with 20 mL ice‐cold artificial cerebrospinal fluid (ACSF, oxygenated with 95% O_2_ and 5% CO_2_) containing (in mm) 210 sucrose, 125 NaCl, 2.5 KCl, 25 NaHCO_3_, 1.25 NaH_2_PO_4_, 1 MgCl_2_, 1 CaCl_2_, 25 glucose, and 1 sodium pyruvate. Sagittal brain slices (300 µm thickness) were sectioned in cold ACSF with a Leica VT1200S vibratome and immediately transferred to an incubation chamber with ASCF containing (in mm) 125 NaCl, 2.5 KCl, 25 NaHCO_3_, 1.25 NaH_2_PO_4_, 1 MgCl_2_, 1 CaCl_2_, 25 glucose. Slices were allowed to recover at 32 °C for 30 min and then transferred to room temperature for 1 h before recording.

### In Vitro Electrophysiology

Whole‐cell patch‐clamp recordings were performed from neurons in the CA1 brain region and the slices were placed in recording chamber perfused with gassed ACSF containing (in mm) 125 NaCl, 2.5 KCl, 25 NaHCO_3_, 1.25 NaH_2_PO_4_, 1 MgCl_2_, 1 CaCl_2_, 25 glucose, which is heated with automatic temperature controller (28–30 °C, TC‐324C, Warner Instruments). Slices were continuously perfused at 2–3 mL min^−1^ with the ACSF. Whole‐cell recording patch pipettes (5–6 MΩ) were pulled with pipette puller (PC‐100, Narishige) from borosilicate glass (Sutter Instrument). Data were acquired with a MultiClamp 700B amplifier and pCLAMP 10.6 software (Axon Instruments), filtered at 2 kHz, and digitized at 10 kHz using Digidata 1550B. The series resistance and capacitance were compensated automatically after a stable Gigaseal was formed. Experiments were excluded if the series resistance was fluctuated >20% from the initial value.

For sEPSC recording, patch pipettes were filled with internal solution containing (in mm) 127 K‐gluconate, 13 KCl, 4 Mg3‐ATP, 0.3 Na3‐GTP, 0.3 ethylene glycol‐bis(*b*‐aminoethylether)‐*N*,*N*,*N9*,*N9*‐tetraacetic acid (EGTA), 10 *N*‐(2‐hydroxyethyl)piperazine‐29‐(2‐ethane‐sulfonic acid) (HEPES), and 10 Na‐phosphocreatine (pH = 7.25). For sIPSC recording, patch pipettes were filled with internal solution containing (in mm) 125 Cs‐methanesulfonate, 8 NaCl, 2 Mg‐ATP, 0.3 Na3‐GTP, 0.3 EGTA, 10 HEPES, and 10 Na‐phosphocreatine (pH = 7.25).

To evoke AMPAR EPSCs in CA1 pyramidal cells, a bipolar stimulation electrode was placed in CA3–CA1 Schaffer collateral synapses and the cell was held at −70mV while repetitive stimulation was applied at 0.1 Hz. For LTP induction, one train of high‐frequency stimulation (100 Hz, 1s) separated by 20 s was performed. To avoid “washing out” of LTP, the induction protocol was applied within 10 min after achieved whole‐cell configuration. The paired‐pulse ratio was valued as the ratio of the amplitude of the second evoked EPSC to the first one.

Offline electrophysiological data were analyzed with Clampfit 10.6 (Molecular Devices) and Mini Analysis Program (Synaptosoft Inc., NJ).

### Plasmid Constructs and Viruses

For AAV vectors, AAV2/9‐CMV–*YTHDF2*–flag–mCherry–WPRE–SV40, AAV2/9‐CMV–GFP–WPRE, AAV2/9‐H1–shRNA (*SEMA4B*)–CAG–eGFP–WPRE–pA were all designed and constructed by standard methods as previously reported.^[^ ^11^ ^]^ AAV viruses were prepared by Taitool Biotech (Shanghai). *SEMA4B* shRNA target sequence: CCACCCATCTTCTAATTCCTA.

### Intrahippocampus Microinjections

Mice were anesthetized with pentobarbital sodium (60 mg kg^−1^, i.p.) and then mounted in a stereotaxic apparatus (RWD Life Science, China). Small bilateral holes were drilled into the skull at −1.7 mm posterior and −1.5 mm lateral to bregma for injections into the hippocampal CA1 and DG regions. Glass cannula filled with a virus solution was lowered to CA1 (−1.5 mm) and DG (−2.0 mm), and the virus solution (1.0 µL) was injected using a Nanoject II (Drummond) system at a rate of 0.1 µL min^−1^ sequentially into each side of hippocampus. Upon completion of the injection, the injectors were left in place for additional 5 min to assure adequate diffusion of the solution. The scalp was then sealed and injected mice were monitored as they recovered from anesthesia. Behavioral experiments or electrophysiological recordings were performed at least 14 days after virus injection. Virus infection was examined at the end of all the behavioral tests.

### Sparse Labeling and Spine Density Analysis

For sparse labeling of pyramidal neurons in hippocampus, NCSP‐YFP‐2E5 (BC‐SL001) was ordered from BrainCase Biotech (Wuhan, China). A glass cannula filled with the viral solution was lowered to a depth of dorsoventral −1.7 mm, and 0.2 µL of the virus was injected at a constant rate to achieve sparse labeling of dendritic spines. Two weeks postinjection, mouse brains were collected, sectioned, and imaged using a 100× oil immersion objective on an Olympus BX53 upright microscope. Apical dendrites within the stratum radiatum of the CA1 region were selected for quantification. For quantitative spine density analysis, images were analyzed in a blind manner. Spines were manually counted along tertiary dendritic segments using ImageJ software.

### RNA‐Seq

TRIzol reagent (Invitrogen, Cat#15596018) was used to extract the total RNA from control and DF2‐CKO mouse hippocampus following the manufacturer's instructions. Three or four biological replicates were performed for each genotype. Total RNA was subjected to BGISEQ sequencing platform by Beijing Genomics Institute (BGI; Wuhan, China). At least 40 million clean reads of sequencing depth were collected for each sample. RNA‐seq raw data were initially filtered to obtain clean data after quality control, and the clean data were aligned to the mouse genome (GCF_000001635.26_GRCm38.p6 by HISAT and Bowtie2. DEseq2 was used for DEG analysis.

### KEGG and GO Enrichment

GO and KEGG analyses were performed using clusterProfiler9 package in R software.

### *YTHDF2*‐RIP‐Seq

Wild‐type mice hippocampus were isolated at 1 h after fear conditioning and snap frozen in liquid nitrogen. Four pairs of hippocampus were used for each biological replicate. Two biological replicates were performed. For each replicate, tissues were collected and frozen in liquid nitrogen, and grinded by tissue lyser. The grinded powder was treated with cell lysis buffer. The 10% lysis sample was stored and named “input,” and 80% was used in immunoprecipitation reactions with anti‐*YTHDF2*‐antibody‐conjugated protein A/G magnetic beads and named “IP,” and 10% was incubated with rabbit IgG (Cell Signaling Technology) as a negative control and named “IgG,” respectively. The RNA of input and IP was extracted using TRIzol reagent. The stranded RNA sequencing library was constructed by using KC‐Digital Stranded mRNA Library Prep Kit for Illumina (Cat#DR08502, Wuhan Seqhealth Co., Ltd. China) following the manufacturer's instruction. The library products corresponding to 200–500 bps were enriched, quantified, and finally sequenced on NovaSeq6000 platform (Illumina, USA) with PE150 model at the LC‐BIO Bio‐tech ltd. (Hangzhou, China) following the vendor's recommended protocol.

Identified *YTHDF2* binding peaks with a bed or bam format was adapted for visualization on the IGV software (http://www.igv.org/).

### RNA Decay Test

The rate of RNA decay was tested by application of transcriptional inhibitors ActD (Sigma‐Aldrich, SBR00013). Actinomycin D (5 µg mL^−1^) was added to cultured primary hippocampus neurons at 6, 3, and 0 h before collection. The total RNA was extracted with TRIzol reagent and subjected to RT‐qPCR analysis or DNBSEQ sequencing platform by BGI (Wuhan, China). One well of a six‐well dish represented one biological replicate and three biological replicates were performed for each time point.

RNA half‐life was calculated as previously described.^[^ ^12^ ^]^ The degradation rate of RNA *k* was estimated by log_2_(*A_t_*/*A*_0_) = −*kt*, where *t* is the transcription inhibition time (h), *A_t_* and *A*_0_ represent the mRNA quantity (attomole) at time *t* and time 0. Two *k* values were calculated: time 3 versus 0 h, and time 6 versus 0 h. The final lifetime was calculated by using the average of *k*_3h_ and *k*_6h_, *t*_1/2_ = 2/(*k*_3h_ + *k*_6h_).

### Proteomics

Control and DF2‐CKO mice hippocampus were isolated at basal level or 4 h after fear conditioning and flash‐freezing in liquid nitrogen. Three biological replicates were performed for proteomics. 4D LFQ proteomics analysis, including trypsin digestion, liquid chromatography–tandem mass spectrometry (MS/MS) analysis, and data analysis were provided by Jingjie PTM Bio‐Labs. After tryptic digestion, peptide samples were dissolved in 2% of acetonitrile/0.1% of formic acid. The peptides were separated using a nanoElute UHPLC system (Bruker Daltonics, Billerica, MA, USA) and subjected to a capillary source followed by timsTOF Pro (Bruker Daltonics, Billerica, MA, USA) mass spectrometry. The resulting MS/MS data were processed using MaxQuant search engine (v.1.6.6.0). Tandem mass spectra were searched against SwissProt Mouse concatenated with a reverse decoy database. To determine differentially expressed proteins, the fold change was calculated as the average of comparisons between biological replicates. Proteins with a fold change >1.2 and *p* < 0.05 were considered to be significantly differentially expressed protein.

### Primary Neuron Culture

Hippocampal neurons derived from postnatal day 0 or 1 (P0/P1) control and DF2‐CKO mice of both sexes were isolated and plated at a density of 200k–300k cells per well on matrigel (Corning)‐precoated 6‐well plates. Neurons were plated in plating medium (neurobasal medium supplied with 0.5 mm Glutamax, 2% B‐27, and 5% FBS) for 3 h, then changed to complete medium (neurobasal medium supplemented with 0.5 mm Glutamax and 2% B‐27) for further culturing. Half media changes were conducted 3 times per week. Neurons were collected and assayed at days 13–14.

### In Vitro SUnSet

A protein translation assay was performed as previously described using the SUnSET method.^[^ ^27^ ^]^ This technique assessed, via western blot, the levels of puromycin incorporation into elongating peptide chains, and was widely used to measure active in vivo translation. Potassium chloride depolarization (KCl, 50 mm) was used to induce noticeable protein synthesis in neurons.^[^ ^11^ ^]^ The primary neurons cultured in 6 well‐plates were treated with 50 mm KCl for 10 min before the complete culture medium was changed back. 2 h after the KCl treatment, puromycin (Gibco, A1113803) was added to the culture media treatments to a final concentration of 1 µm for 30 min, then the neurons were washed 2× with PBS and lysed. The nascent proteins were immunoprecipitated with an anti‐puromycin monoclonal antibody (1:5000; Millipore; MABE343).

### Ribosome Profiling

Ribosome profiling (Ribo‐seq) was used to analysis the translation activity in DF2‐CKO mice. Control and DF2‐CKO mice hippocampus were isolated at 1 h after fear conditioning. Four pairs of hippocampus were used for each biological replicate. Three biological replicates were performed for ribosome profiling. The hippocampus tissues were cut into rice grain size and treated with 100 µg mL^−1^ cycloheximide and then flash‐freezing in liquid nitrogen. Ribosome profiling was performed using Epi Ribosome Profiling Kit (Epibiotek, R1814). Ribosome‐protected RNA fragments were extracted using RNA clean&ConcentratorTM‐5 kit (ZYMO, R1016). Epi RiboRNA Depletion Kit (Human/Mouse/Rat) (Epibiotek, R1805) was used for rRNA depletion. Sequencing libraries were constructed using QIAseq miRNA Library kit (QIAGEN, 1103679), and sequenced with Illumina Novaseq 6000, following the protocol from company (Epibiotek, Guangzhou). For the total RNA controls, the total RNA was extracted with TRIzol reagent and VAHTS Stranded mRNA‐seq Library Prep Kit for Illumina V2 (Vazyme Biotech, NR612‐02) was used for library preparation.

For Ribo‐seq data processing, Adapter sequences were removed from raw sequencing data using cutadapt software. Meanwhile, reads with length between 25 and 35 bp were kept for downstream analysis. Then, reads were aligned to rRNA and tRNA sequences so as to remove rRNA and tRNA reads using bowtie software, remaining reads were used to align to reference genome and transcriptome (Ensembl Version 91) using hisat2 and bowtie software separately. The trinucleotide periodicity of ribosomes and codon usage frequency were estimated using revised riboWaltz package. ORF identification was performed using Price software. Read counts were calculated using feature Counts software. Raw counts were further normalized as RPKM values using fpkm function in DESeq2 package. Translational efficiencies were determined as the ratio of (normalized abundance determined by ribosome profiling)/(normalized abundance determined by RNA‐seq) as previously reported.^[^ ^12^ ^]^

### qPCR

Total RNA was extracted from hippocampus using TRIzol Reagent, and the procedures were conducted following the manufacturer's instructions. The reverse transcription into cDNA libraries was carried out according to the protocol of the cDNA synthesis kit (Vazyme, Cat# R212‐02), and qPCR was conducted using Universal SYBR qPCR master mix (Vazyme, Cat# Q511‐02). The assays were performed in a 96‐well reaction plate on a Quant Studio 3.0 thermocycler (Applied Biosystems, USA). The ratio of mRNA expression was determined using the 2‐ΔΔCT method. The specific primer sequences used for qPCR were as follows: Primer: *YTHDF2*, Forward: GCTTGCCTGCTACATAGTGAGA, Reverse: AACTGAACTGCTTAACCTTCTGG; Primer: *BDNF*, Forward: GGCTGACACTTTTGAGCACGTC, Reverse: CTCCAAAGGCACTTGACTGCTG; Primer: *GRIN2B*, Forward: TCCGAAGCTGGTGATAATCC, Reverse: CTTCTGGCACGGGACTGTAT; Primer: *SLITRK1*, Forward: TTGGACCTCAGGTGGCTCTACA, Reverse: GGAGAATGAGCTGAATCGCGTTG; Primer: *SLITRK2*, Forward: GCAGAGCTTGCAGTACTCTCTATT, Reverse: GGACCTCCAGCAGGTTGTTATT; Primer: *SHANK2*, Forward: CCTCCAGGACTGCAGAGAAC, Reverse: ATTTCTGCCTTCGCATCGTA; Primer: *SEMA4B*, Forward: GGCTACAAGACCCTGCCTTT, Reverse: CACCCTCATCGCCCTTACAG.

### PSD Preparation

PSD fraction was prepared as previously described.^[^ ^11^ ^]^ Briefly, hippocampal tissues were homogenized in homogenization buffer (320 mm sucrose, 5 mm sodium pyrophosphate, 1 mm ethylenediaminetetraacetic acid (EDTA), 10 mm HEPES (pH 7.4), 1× protease inhibitor cocktail, and 1× phosphatase inhibitor cocktail (Sigma). The resultant homogenate was centrifuged at 800*g* for 10 min at 4 °C to yield postnuclear pelleted fraction 1 (P1) and supernatant fraction 1 (S1). S1 was further centrifuged at 15 000*g* for 20 min at 4 °C to yield pelleted fraction 2 (P2). Then, pellet P2 (which contains the synaptosome) was resuspended in 4 mm HEPES (pH 7.4) and incubated with agitation at 4 °C for 30 min. Suspended P2 was centrifuged at 25 000*g* for 20 min at 4 °C. The resulting pellet was resuspended in 50 mm HEPES (pH 7.4), mixed with an equal volume of 1% Triton X‐100, and incubated with agitation at 4 °C for 15 min. The PSD fraction was generated by centrifugation at 32 000*g* for 20 min at 4 °C. The final PSD pellet was resuspended in 50 mm HEPES followed by protein quantification and then boiled with 6× loading buffer for western blot.

### *YTHDF2* RIP with RT‐qPCR

RIP‐qPCR was performed as previously described,^[^ ^53^ ^]^ with minor modifications. The assay was conducted using the EZ‐Magna RIP RNA‐Binding Protein Immunoprecipitation Kit (Millipore, 17‐701) according to the manufacturer's instructions. Hippocampal tissue was homogenized in complete RIP lysis buffer supplemented with a protease inhibitor cocktail, and incubated on ice for 60 min at 4 °C. The lysates were cleared by centrifugation at 18 000 × *g* for 20 min at 4 °C. Immunoprecipitation was then performed using a rabbit anti‐*YTHDF2* antibody (Proteintech, 24744‐1‐AP) or control rabbit IgG. Ten percent of the cleared lysate was reserved as the input. RNA was extracted from both the immunoprecipitated samples and input, and then subjected to reverse transcription followed by quantitative PCR. As a negative control, the conserved ribosomal gene *RPS29*, which showed no binding to *YTHDF2* in our RIP‐seq data, was included in the RT‐qPCR analysis. Enrichment of target transcripts was calculated as % Input, taking into account both the *C*_t_ values and the volume differences between IP and input samples. Data represented results from two independent biological replicates.

### MeRIP Combined with RT‐qPCR

MeRIP‐qPCR was performed to quantify m^6^A modifications on target mRNAs.^[^ ^53^ ^]^ Total RNA was extracted from cultured primary hippocampus neurons using TRIzol reagent (Invitrogen, 15596026) following the manufacturer's instructions. For each IP reaction, 33 µg of total RNAs was fragmented by adding RNA fragmentation buffer (Invitrogen, AM8740) and incubating for 3 min at 70 °C. The fragmented RNAs were then recovered using ethanol‐precipitation‐based method with three volumes of ethanol, one‐tenth volume of 3 m sodium acetate, and 1 µL of glycogen (Thermo Fisher Scientific, R0551). Subsequently, the fragmented RNAs were subjected to IP using an anti‐m^6^A antibody (Millipore, ABE572) or a control IgG antibody (Millipore, PP64) along with protein‐A&G magnetic beads (Thermo Fisher Scientific, 88803) in IP buffer [50 mm tris‐HCl (pH 7.4), 150 mm NaCl, and 0.5% IGEPAL CA‐630, supplemented with RNase inhibitor] for 2 h at 4 °C. One‐tenth of the fragmented RNAs were saved as “input.” Following IP, the beads were washed with IP buffer, and the bound RNAs were released by incubation in proteinase K reaction buffer [100 mm tris‐HCl (pH 7.4), 50 mm NaCl, 1 mm EDTA, 0.2% SDS, and proteinase K (0.95 mg mL^−1^)] for 30 min at 55 °C. The released RNAs were then recovered using ethanol‐precipitation‐based method. The RNAs obtained from the IP, as well as the input RNAs, were used for subsequent RT‐qPCR analysis. A conserved ribosomal gene *Rps21*, which showed no m^6^A modification in meRIP‐seq data,^[^ ^53^ ^]^ was selected as negative control. Three biological replicates were performed for each group. No PCR amplification was detected in the negative IP samples using the control IgG antibody.

### Quantification and Statistical Analysis

All statistical analysis and graph construction were done in GraphPad Prism and R studio. Results are presented as mean ± standard error of mean. Statistical significances were assessed using GraphPad Prism using either unpaired two‐tailed *t*‐tests to compare values between two specific groups or one‐way ANOVA followed by Tukey's/Bonferroni post‐hoc test to compare the values of more than two groups or two‐ANOVA with Bonferroni post‐hoc test to compare two or more than two groups at a particular time point or repeated measures two‐way ANOVA with Bonferroni multicomparison test to compare the values of two or more than two groups at different time points. In the event of non‐Gaussian distribution data, the Mann–Whitney test, Kruskal–Wallis test followed by Dunn's multiple comparison test or Uncorrected Dunn's test, or Kolmogorov–Smirnov test were employed. Notably, for sEPSC and sIPSC recordings, considering the heterogeneity of different mice, linear mixed effect analyses were used, in which individual was treated as a random effect.^[^ ^54^ ^]^ All statistical tests, values, and significance levels were specified in the associated figure legends. In violin plots, the median and quartiles were depicted as lines, while in bar plots, the error bars represented the mean with standard error. The *p* value of 0.05 or less was considered statistically significant in all statistical analyses.

### Data Availability

All the processing data of RNA sequencing and proteomics were provided as Tables S1–S5 (Supporting Information), including all the expression abundance of RNA/protein and following analysis. The authors declared that all other data supporting the findings of this study were available within the paper and its Supporting Information files.

### Code Availability

Custom codes were available from the corresponding author upon request.

## Conflict of Interest

The authors declare no conflict of interest.

## Author Contributions

K.L., C.G., X.W., C.W. contributed equally to this work. K.L., Y.C., and T.Z. conceived the project; K.L. and T.Z. designed the experiments and wrote the paper with the input from Y.C.; C.G. and Y.C. contributed to all the electrophysiological recordings and related data analysis. X.W. contributed to the behavior test and neurogenesis analysis; C.W. and S.W. contributed to BrdU labeling, Ki67 analysis, and meRIP‐qPCR; S.S., M.W., X.Z., and S.L. contributed to mice breeding and all other experiments. All authors helped prepare the paper.

## Supporting information



Supporting Information

Supplemental Table 1

Supplemental Table 2

Supplemental Table 3

Supplemental Table 4

Supplemental Table 5

## Data Availability

The data that support the findings of this study are available from the corresponding author upon reasonable request.
