# Supplementary material for: Enhanced Protein Synthesis and Hippocampus‐Dependent Memory via Inhibition of YTHDF2‐Mediated m6A mRNA Degradation
Source: Adv Sci (Weinh). 2025 Sep 17;12(45):e14926. doi: 10.1002/advs.202514926 (PMC12677670; doi:10.1002/advs.202514926)
Supplement: Supplementary file 1 — Supporting Information [file ADVS-12-e14926-s006.docx]

**Supplemental material**

**Supplemental methods**

**Open-field test.**

Mice were exposed to a square open test chambers (40 cm × 40 cm) with opaque base and walls (40 cm high). Each mouse was individually placed in the center of the chamber and allowed 20 min to explore the area and its activity was recorded and analyzed using the JLBehv- LAM-4 (JiLiang, Shanghai). The surfaces of chambers were cleaned with 70% ethanol after each mouse was tested.

**Elevated-plus maze.**

Elevated-plus maze apparatus consists of two open arms (26 cm ×6 cm), two enclosed arms (26 cm × 6 cm × 14.5 cm) and a central area (6 cm × 6 cm). The maze is elevated 70 cm above ground in a room with normal light. Mice were placed in the central region of the maze and their position was tracked for 5 min. The time of each mouse spent in the open arms and open arm entries during the 5-min exploration was counted by JLBehv-EPMM-4 (JiLiang, Shanghai).

**Light-dark box transition test.**

The light-dark transition box (LDB) test consists of a two-compartment box (black and dark, 25 × 20 × 30 cm; white and illuminated 400 lux, 25 × 30 × 30 cm) connected by a hole (7 × 7 cm). During the test, mice were individually placed at the center of the light compartment facing away from the hole and allowed 10 min to explore freely in the box. The time mice spent in the light compartment, transition times as well as the total distance moved were automatically calculated by JLBehv-PAM-2 (JiLiang).

**Rota-Rod test**

Rota-Rod test was used to evaluate the sensorimotor coordination of mice. Animals were habituated to the Rota-Rod and trained to remain on a rotating drum (constant speed of 6 r.p.m.) for a minimum of 90 s for 2 consecutive days. In the testing sessions, animals were placed on the Rota-Rod and tested for a maximum of 300 seconds each. The initial velocity of the rod was 4 RPM, and was accelerated every 10 seconds by 4 RPM until reaching 40 RPM. The amount of time spent on the rod before falling (latency time to fall) was recorded for each animal.

**BrdU labeling**

The proliferation capacity of DF2-CKO hippocampus was assessed using BrdU, an analog of thymidine that labels proliferating cells by incorporating into synthesizing DNA during the S phase of the cell cycle. For in vivo labeling of proliferating cells at different developmental stages, BrdU (Sigma-Aldrich, B5002) was intraperitoneally injected at a dose of 100 mg/kg body weight into pregnant mice at embryonic day 17.5 (E17.5) or into postnatal pups at P3, P14, and P30. For each time point, brains were collected 2 hours after BrdU injection, followed by fixation, dehydration, and sectioning for subsequent immunohistochemistry (IHC). Antigen retrieval was performed by immersing the sections in citrate buffer (Sangon Biotech, E673000) at 95°C for 15 min, followed by incubation at RT for 60 min. Subsequently, the sections were washed twice with PBS and treated with 2 M HCl for 30 min, followed by another three washes with PBS. The samples were then subjected to the standard immunostaining procedure, including blocking and antibody incubation. An anti-BrdU antibody (Abcam, ab6326, 1:1000 for IHC) was used to detect the incorporated BrdU.

**Golgi staining and** **spine analysis**

The FD Rapid GolgiStain Kit^TM^ (FD NeuroTechnologies, Columbia, MD, USA) was used according to the manufacturer’s instructions. Briefly, mice were subjected to fear conditioning and euthanized 24 hours later, and the brains were rapidly removed. After a brief rinse with double distilled water, the brain was immersed in the impregnation solution (Solutions A + B, mixed 24 h before use) for 2 weeks in the dark at room temperature. The brains were then transferred to solution C and stored at 4 °C in the dark for 3 days. Brain tissue was sliced at 200 μm using freezing microtome (CM1950, Leica). The sections were mounted on solution C coated glass slides and dried naturally at room temperature. The dry-mounted sections were rinsed with double distilled water for 2 min and then reacted in equal parts of solutions D and E in the dark for 10 min. The sections were further rinsed in double distilled water for 4 min and dehydrated with an ascending series of alcohol baths (50, 75, 95 and 100%). Following dehydration, the sections were cleaned in xylene and finally coverslipped with Permount.

Dendritic spines were examined with a 100× objective oil immersion lens and imaged with a microscope (Olympus BX53 upright microscope). Area CA1 stratum radiatum apical dendrites were quantified. For quantitative analysis of spine density, images were blinded, and spines were manually counted along segments of tertiary dendrites using imageJ.

**Supplemental Figure**


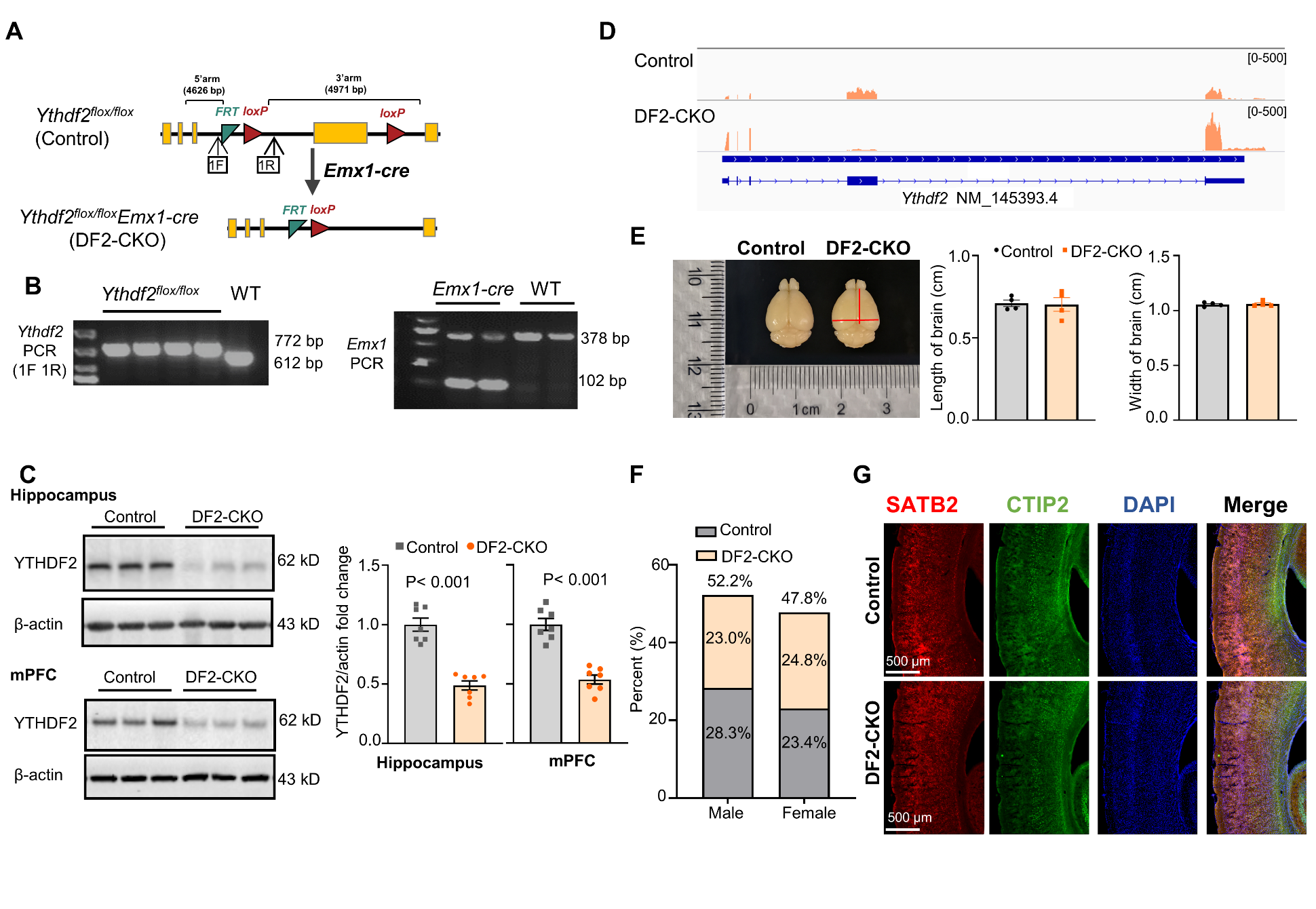


**Supplementary Figure 1. Generation and evaluation of DF2-CKO mice.**

(A) Schematic diagram of the strategy for generating *Ythdf2^fl/fl^Emx1-cre* mice (DF2-CKO) using Cre/loxP recombinase system. *LoxP* sites were designed to target the fourth exon of *Ythdf2*. 1F and 1R, genotyping primers.

(B) Representative genotyping PCR products of mice with different genotypes. The genotyping protocol of *Emx1-cre* mice was sourced from Jackson lab.

(C) Representative blots (left) and quantification data (right) of YTHDF2 expression in mice hippocampus (n = 7 mice/group, unpaired two-tailed t-test, t_12_ = 7.649, p < 0.001) and mPFC. (n = 7 mice/group, unpaired two-tailed t-test, t_12_ = 7.363, p < 0.001).

(D) Visual data of *Ythdf2* sequence in mice hippocampus detected by RNA-seq. The fourth exon of *Ythdf2* was knockout in DF2-CKO mice. Integrative Genomics Viewer was used to display the sequencing data.

(E) Representative images of adult brain from control (left) and DF2-CKO mice (right) and quantification data of brain length (n = 4 mice/group, unpaired two-tailed t-test, t_6_ = 0.164, p = 0.875) and width. (n = 4 mice/group, unpaired two-tailed t-test, t_6_ = 0.233, p = 0.824).

(F) Offspring mice from control and DF2-CKO groups were produced following the strategy outlined in (A) (total n = 100 mice). The expected distribution of control and DF2-CKO mice was around 50% for each group.

(G) Representative confocal immunostaining of CTIP2 (a marker for deep layer cortical neurons) and SATB2 (a marker for upper layer cortical neurons) in the cortex of adult control and DF2-CKO mice.


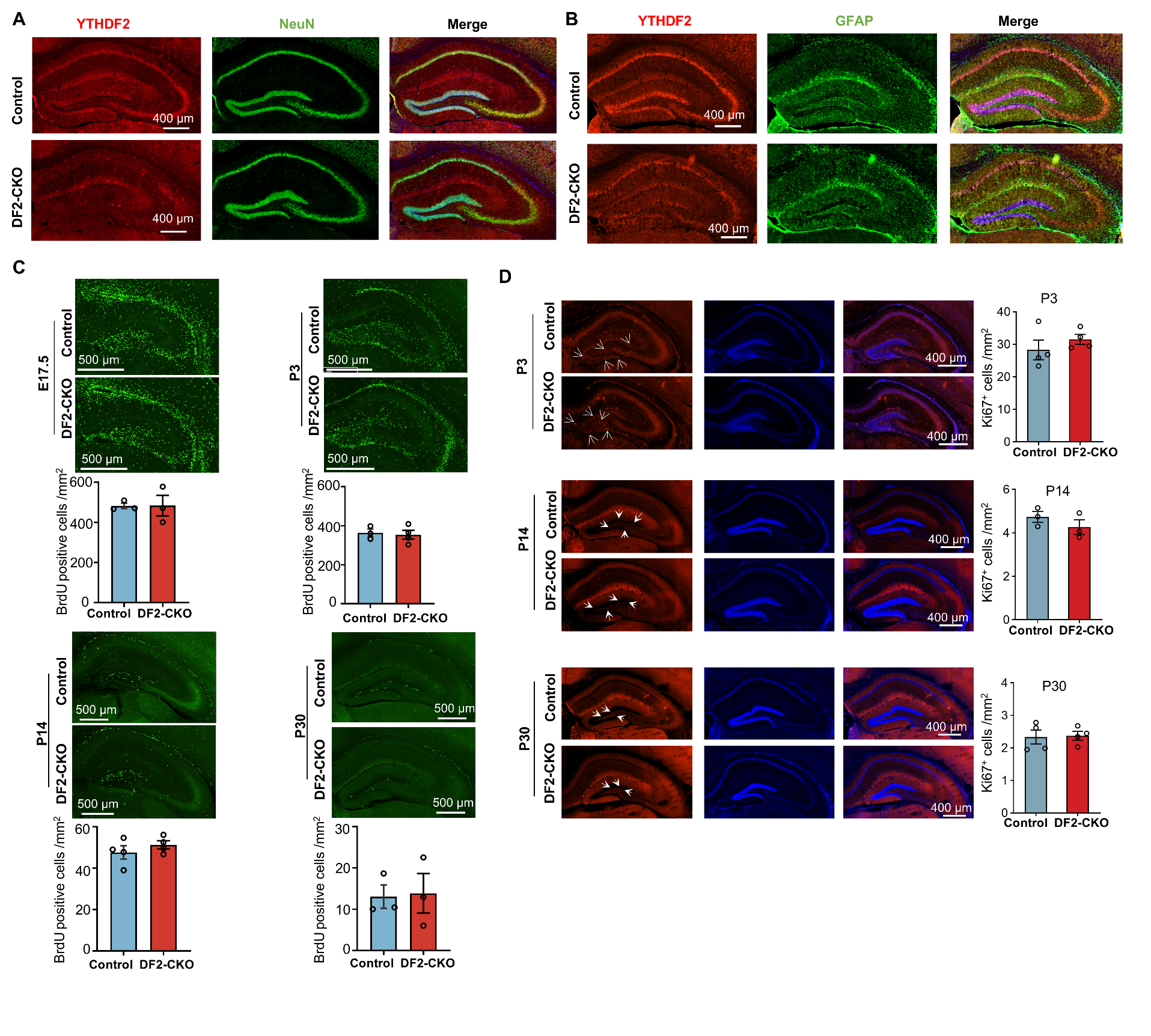


**Supplementary Figure 2. The hippocampus of DF2-CKO mice displayed normal morphology and proliferative activity.**

(A) Representative confocal immunostaining of YTHDF2 and NeuN (a marker for neuron) in the hippocampus of adult control and DF2-CKO mice.

(B) Representative confocal immunostaining of YTHDF2 and GFAP (a marker for astrocyte) in the hippocampus of adult control and DF2-CKO mice.

(C) Assessment of cell proliferation through BrdU labeling on hippocampus from Control and DF2-cKO mice at E17.5, P3, P14, and P30. The proliferative activity was assessed by counting the number of BrdU positive cells per unit area (mm²) within unilateral hippocampus. Each data point in the bar graph represents the mean value from the unilateral hippocampus of a single mouse (E17.5: n=3 mice/group, unpaired two-tailed t-test, t_4_ = 0.022, p = 0.984; P3: n=3, 4 mice, t_5_ = 0.316, p = 0.765; P14: n=4 mice/group, t_6_ = 953, p = 0.378; P30: n=3 mice/group, t_4_ = 0.143, p = 0.893).

(D) Representative immunofluorescence images and quantification of Ki67-positive cells in the hippocampus at P3, P14, and P30. The proliferative activity was assessed by counting the number of Ki67-positive cells per unit area (mm²) within unilateral hippocampus. Each data point in the bar graph represents the mean value from the unilateral hippocampus of a single mouse (P3: n=4 mice/group, unpaired two-tailed t-test, t_6_ = 0.965, p = 0.372; P14: n=3 mice/group, unpaired two-tailed t-test, t_4_ = 1.124, p = 0.324; P30: n=4 mice/group, unpaired two-tailed t-test, t_6_ = 0.168, p = 0.872).


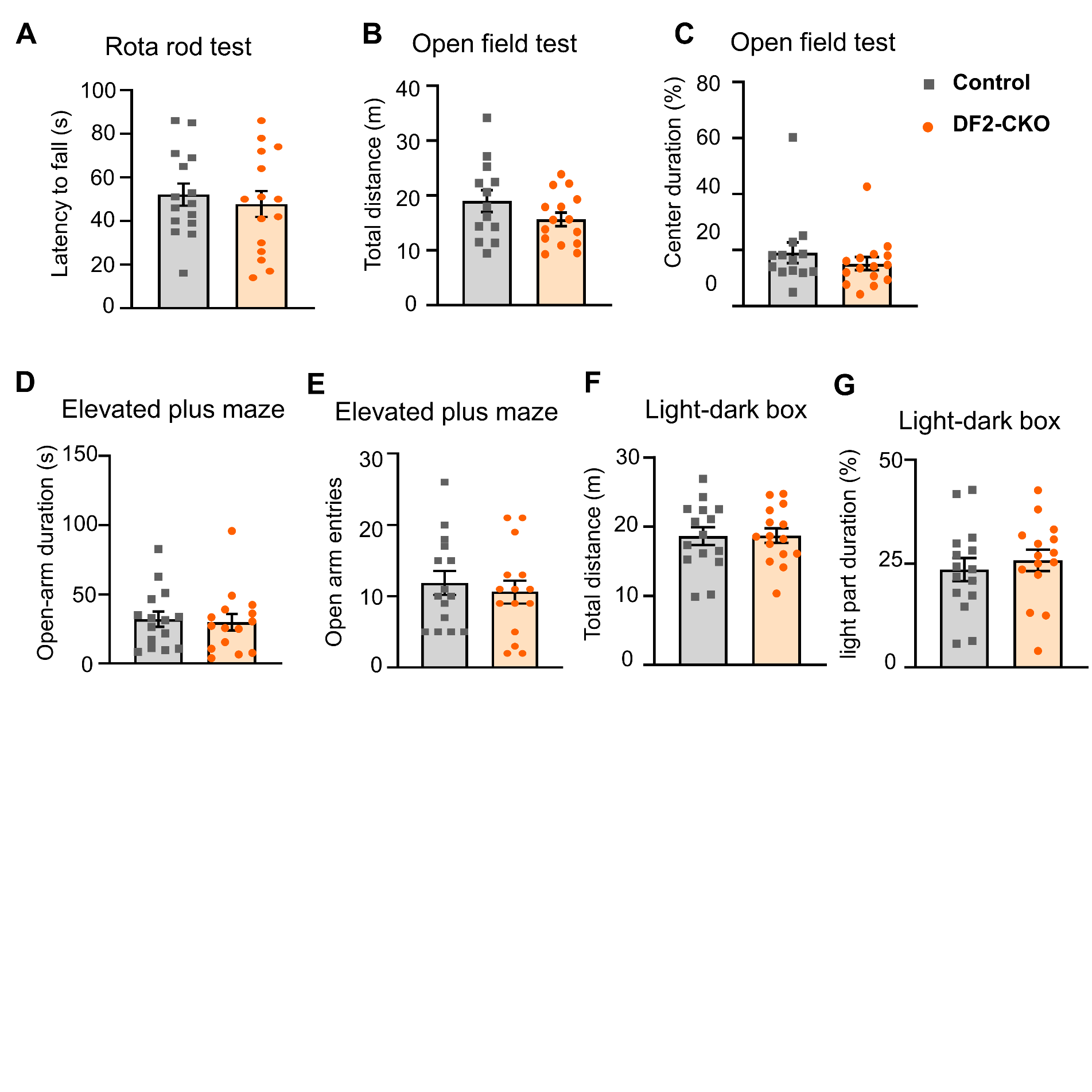


**Supplementary Figure 3. The locomotor activities and anxiety-like behaviors were not affected in DF2-CKO mice.**

(A) Time spent on the rotating rod of control and DF2-CKO mice. (n = 15 mice/group, unpaired two-tailed t-test, t_28_ = 0.543, p = 0.592).

(B, C) Total distance moved (B) and percentage of time spent in central area (C) of control and DF2-CKO mice during 5 min intervals in open field test. (B: n = 13,15 mice, unpaired two-tailed t-test, t_26_ = 1.479, p = 0.151; C: n = 13,15 mice, unpaired two-tailed t-test, t_26_ = 0.914, p = 0.369).

(D, E) Time spent in open arm (D) and open arm entries (E) of control and DF2-CKO mice during 5 min intervals in elevated plus maze. (D: n = 15 mice/group, unpaired two-tailed t-test, t_28_ = 0.273, p = 0.787; E: n = 15 mice/group, unpaired two-tailed t-test, t_28_ = 0.548, P = 0.588).

(F, G) Total distance moved (F) and percentage of time spent in light box (G) of control and DF2-CKO mice during 10 min intervals in light-dark box. (F: n = 15 mice/group, unpaired two-tailed t-test, t_28_ = 0.042, p = 0.967; G: n = 15 mice/group, unpaired two-tailed t-test, t_28_ = 0.587, p = 0.562).


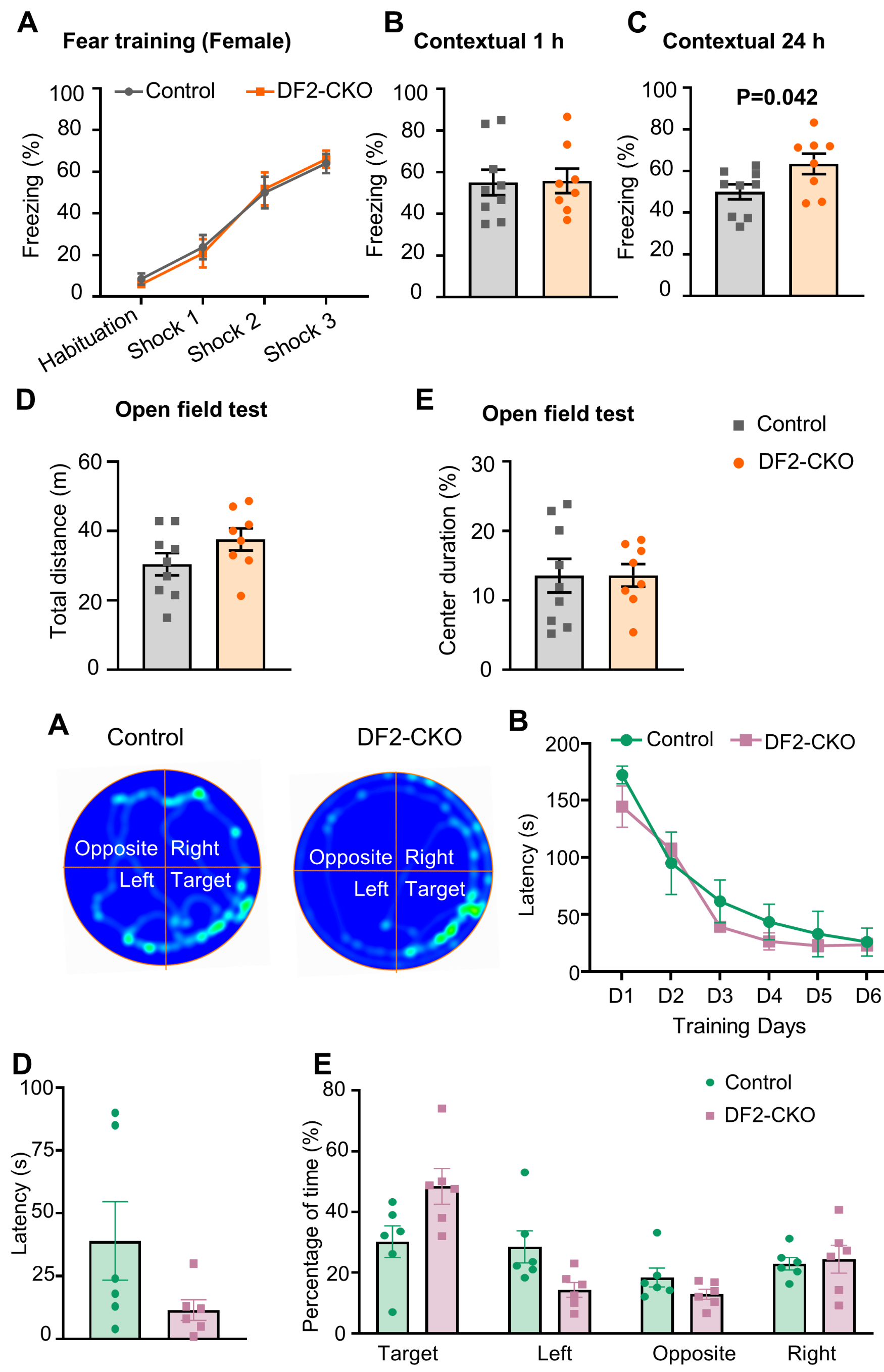


**Supplementary Figure 4. Fear memory evaluation in female DF2-CKO mice.**

(A) The freezing curves of female control and DF2-CKO mice during fear conditioning training. (n = 9,8 mice/group, two-way ANOVA, group: F (1, 15) = 0.007, p = 0.936).

(B) Contextual fear memory assessed 1 h after fear training in female control and DF2-CKO mice. (n = 9,8 mice, unpaired two-tailed t-test, t_15_ = 0.084, p = 0.935).

(C) Contextual fear memory assessed 24 h after fear training in female control and DF2-CKO mice. (n = 9,8 mice/group, unpaired two-tailed t-test, t_15_ = 2.226, p = 0.042).

(D, E) Total distance moved (D) and percentage of time spent in central area (E) of female mice during 10 min intervals in open field test. (D: n = 9,8 mice, unpaired two-tailed t-test, t_15_ = 1.572, p = 0.137; E: n = 9,8 mice, unpaired two-tailed t-test, t_15_ = 0.010, p = 0.992).


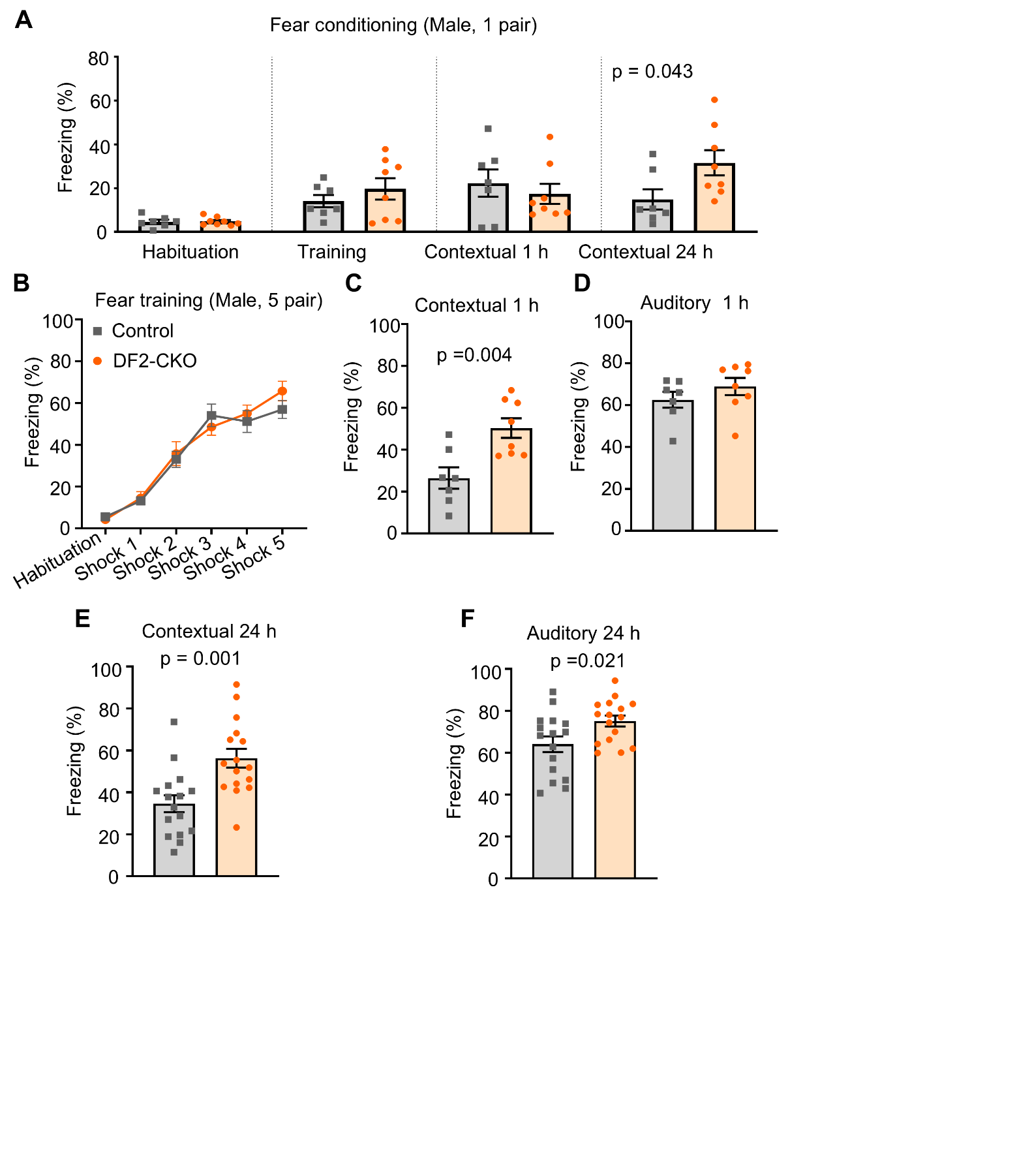


**Supplementary Figure 5. Fear memory evaluation in DF2-CKO mice using weaker and stronger protocols.**

(A) Contextual fear memory assessed under a weaker training protocol (0.8 mA, 1 s, 1 pair) in male control and DF2-CKO mice. (n = 7,8 mice, unpaired two-tailed t-test, Habituation: t_13_ = 0.168, p = 0.869; Training: t_13_ = 0.962, p = 0.354; Contextual 1 h: t_13_ = 0.645, p = 0.531; Contextual 24 h: t_13_ = 2.248, p = 0.043).

(B) The freezing curves of control and DF2-CKO mice during fear conditioning under a stronger training protocol. (0.8 mA, 1 s, 5 pair) (n = 16 mice/group, two-way ANOVA, group: F (1, 30) = 1.136, p = 0.295).

(C, D) Contextual (C) and auditory (D) fear memory assessed 1 h after stronger fear training (C: n = 7, 8 mice, unpaired two-tailed t-test, t_13_ = 3.449, p = 0.004; D: n = 7, 8 mice, unpaired two-tailed t-test, t_13_ = 1.114, p = 0.286).

(E, F) Contextual (E) and auditory (F) fear memory assessed 24 h after stronger fear training. (E: n = 16 mice/group, unpaired two-tailed t-test, t_30_ = 3.608, p = 0.001; F: n = 16 mice/group, unpaired two-tailed t-test, t_30_ = 2.440, p = 0.021).

(G) Performance of control and DF2-CKO mice in the training sessions of Barnes maze test. (n = 6 mice/group, two-way ANOVA, group: F (1, 10) = 0.548, p = 0.476).

(H) Latencies (time to locate the escape box) in the probe trial of the Barnes Maze test. (n = 6 mice/group, unpaired two-tailed t-test, t_10_ = 1.706, p = 0.119).


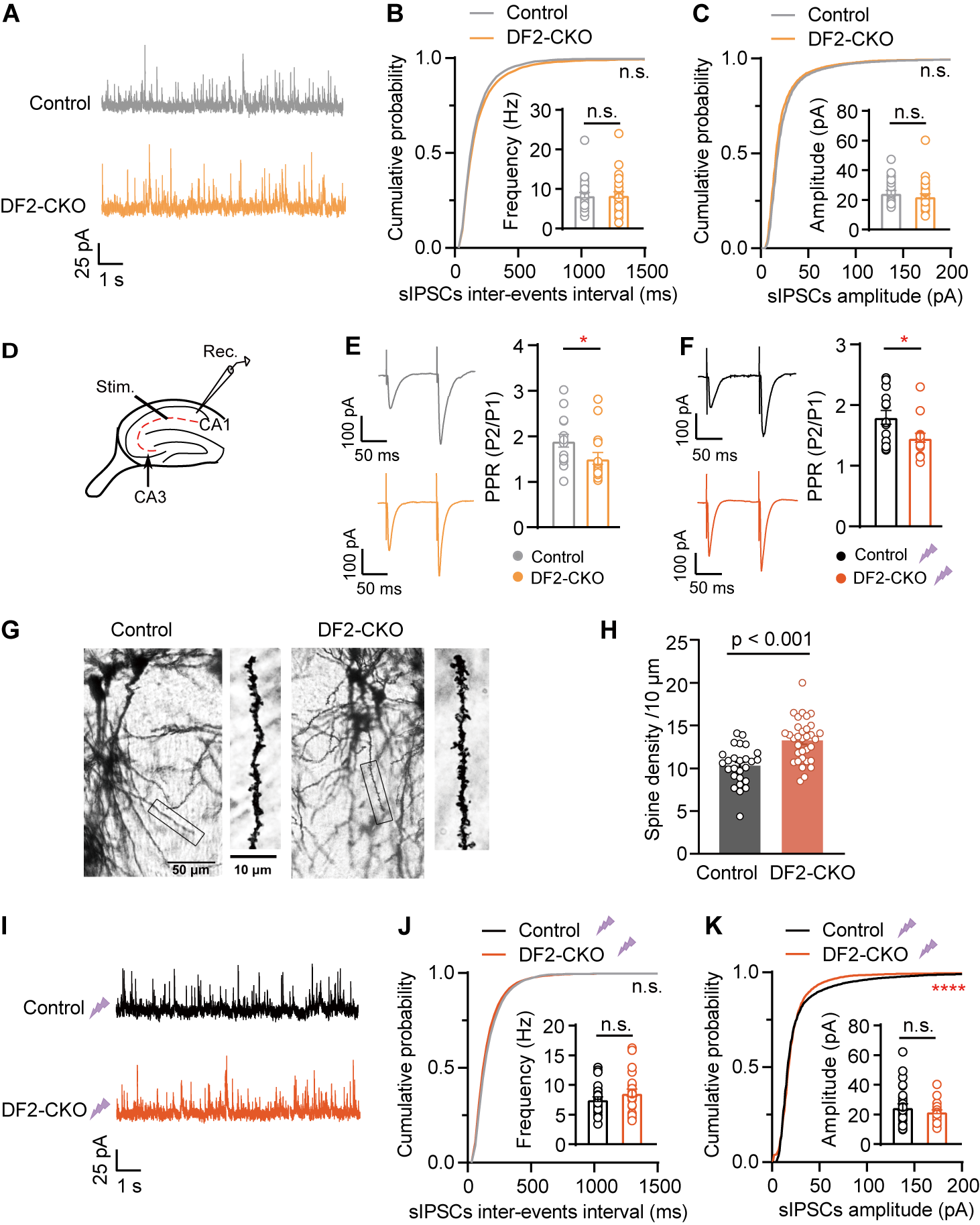


**Supplementary Figure 6. Electrophysiological Records of YTHDF2-depleted hippocampus.**

(A) Representative traces of spontaneous inhibitory postsynaptic currents (sIPSCs) at basal level.

(B-C) Cumulative probability plots and bar graph (inside) showed no change of sIPSCs frequency (B) and amplitude (C) in DF2-CKO mice at basal level. (n_control_ = 31 neurons, n_DF2-CKO_ = 32 neurons; Mann Whitney test, U = 483, p = 0.865, KS test, p > 0.05 for frequency; Mann Whitney test, U = 381, p = 0.116, KS test, p > 0.05 for amplitude).

(D) Schematic diagram of paired-pulse ratio (PPR) recording.

(E-F) Representative traces (left) and histogram (right) of PPR before (E) and after (F) fear conditioning. (E: n_control_ = 15 slices, n_DF2-CKO_ = 16 slices; Mann Whitney test, U = 57, p = 0.012; F: n_control_ = 14 slices, n_DF2-CKO_ = 16 slices; Mann Whitney test, U = 60, p = 0.031).

(G) Representative images of Golgi staining showed the morphology of dendrites and spines of a typical CA1 neuron from either control or DF2-CKO mice at 24 h after fear training.

(H) Statistical analyses of spine density (n = 26 dendrites of 23 neurons from 3 control mice and 33 dendrites of 31 neurons from 3 DF2-CKO mice, unpaired two-tailed *t* test, t_57_ = 4.724, p < 0.001).

(I) Representative traces of spontaneous inhibitory postsynaptic currents (sIPSCs) at 24 h after fear conditioning.

(J-K) Cumulative probability plots and bar graph (inside) showed no change of sIPSCs frequency (J) but a slight decrease of sIPSCs amplitude in DF2-CKO mice after fear conditioning (K). (n_control_ = 33 neurons, n_DF2-CKO_ = 25 neurons; Mann Whitney test, U = 328, p = 0.189, KS test, p > 0.05 for frequency; Mann Whitney test, U = 373, p = 0.543, KS test, p < 0.0001 for amplitude)


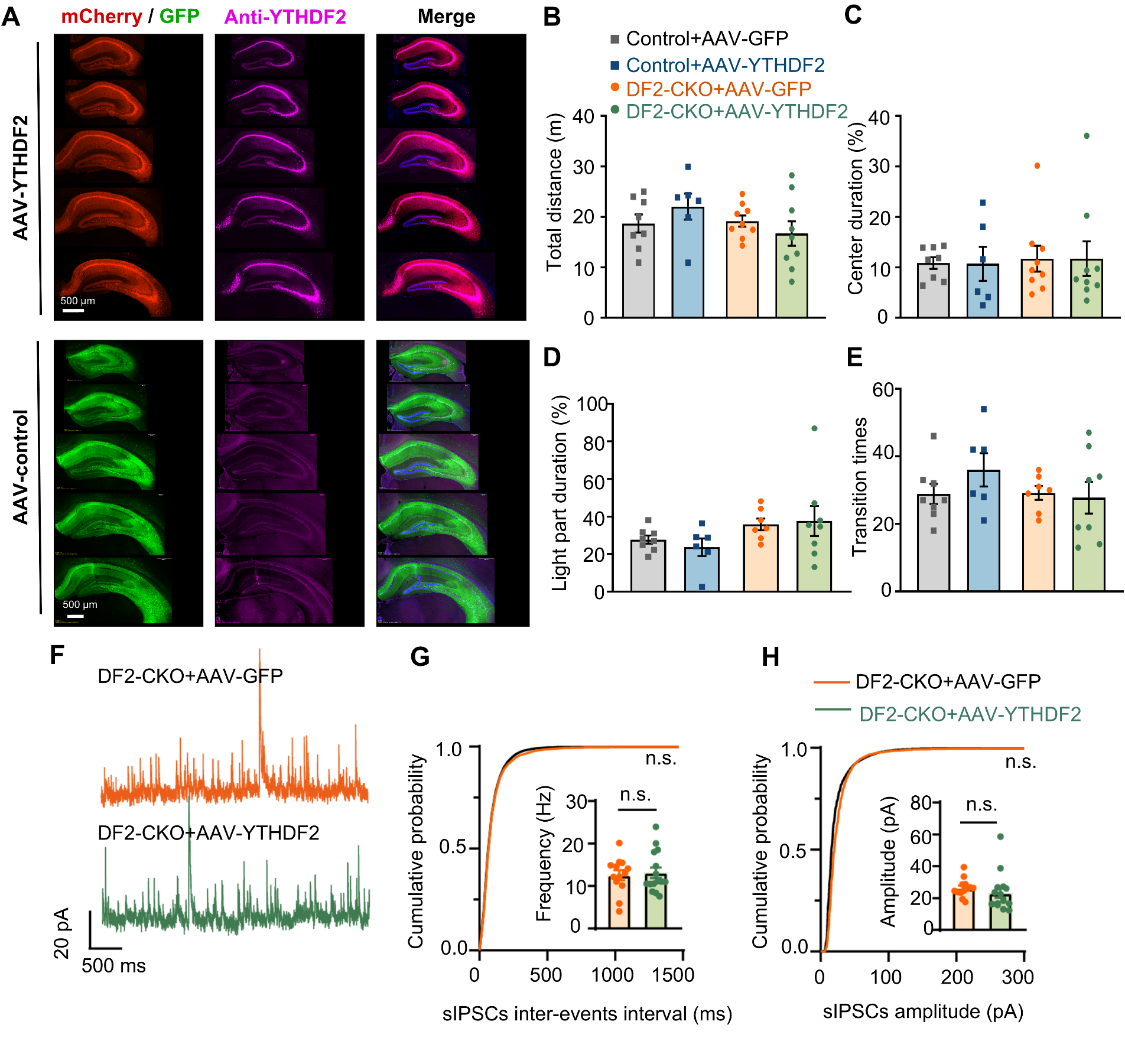


**Supplementary Figure 7. Evaluation of YTHDF2 re-expression in the hippocampus.**

(A) Representative fluorescent images of AAV-YTHDF2-mcherry/AAV- GFP and immunostaining of YTHDF2 (far red) showing the virus injection and YTHDF2 expression. The observed fluorescence signal indicates widespread expression of AAV-YTHDF2 throughout the hippocampus.

(B-C) Total distance moved (B) and the percentage of time spent in central area (C) of mice during 5 min intervals in open field test. (B: n = 8, 6, 9, 9 mice, one-way ANOVA, F_(3, 28)_ = 1.081, p = 0.373; C: n = 8, 6, 9, 9 mice, one-way ANOVA, F_(3, 28)_ = 0.037, p = 0.990).

(D-E) Light part duration (D) and transition times between light part and dark part (E) in Light-Dark box test. (D: n = 8, 6, 7, 8 mice, one-way ANOVA, F_(3, 25)_ = 1.550, p = 0.226; E: n = 8, 6, 7, 8 mice, one-way ANOVA, F_(3, 25)_ = 0.858, p = 0.476).

(F) Representative traces of spontaneous inhibitory postsynaptic currents (sIPSCs).

(G-H) Cumulative probability plots and bar graph (inside) showed unaffected inhibitory transmission in hippocampal CA1 pyramidal neurons from DF2-CKO mice hippocampus injected with AAV-YTHDF2. (n_DF2-CKO+AAV-GFP_ = 13 cells, 2 mice, n_DF2-CKO+AAV-YTHDF2_ = 15 cells, 2 mice; unpaired *t* test, t_26_=0.343, p = 0.734 for frequency; unpaired *t* test, t_26_=0.867, p = 0.394 for amplitude).





**Supplementary Figure 8. The stability of YTHDF2 target mRNAs were increased in DF2-CKO mice.**

(A) Immunoprecipitation efficiency verification of YTHDF2 antibody. YTHDF2 (62 kD) was enriched in IP samples compared to input samples.

(B) Scatterplot showing the correlation of biological replicates in YTHDF2 RIP-seq, based on read counts between different samples.

(C) Bar plot showing the number of significantly upregulated and downregulated mRNAs among YTHDF2 targets and non-targets. Significant differential expression gene was defined as FoldChange (DF2-CKO/Control) >1.2 and p < 0.05.

(D) GO enrichment analysis of YTHDF2 target transcripts with prolonged lifetime (e.g., log_2_FC (DF2-KO/Control halflife) >0.5). Top ten BP and CC terms were displayed.

(E-I) The mRNA levels of *Bdnf* (E), *Grin2b* (F), *Slitrk1* (G), *Slitrk2* (H), and *Shank2* (I) were assessed via RT-PCR in primary cultured neurons from Control and DF2-CKO mice treated with Actinomycin D (ActD) for 0 h, 3 h, and 6 h. (E: n=3 cell dishes/group, repeated two-way ANOVA, group factor, F_(1, 4)_=13.76, p=0.021, post hoc: Bonferroni’s test, 3 h: p=0.041; F: n=3 dishes/group, repeated two-way ANOVA, group factor: F_(1,4)_=70.98, p=0.001, post hoc, Bonferroni’s test, 6 h: p=0.026; G: n=3 dishes/group, repeated two-way ANOVA, group factor: F_(1, 4)_=53.32, p=0.002, post hoc: Bonferroni’s test, 6 h: p=0.003; H: n=3 dishes/group, repeated two-way ANOVA, group factor: F_(1, 4)_=20.77, p=0.010, post hoc, Bonferroni’s test, 3 h: p=0.007; I: n=3 dishes/group, repeated two-way ANOVA, group factor: F_(1, 4)_=5.497, p=0.027, post hoc: Bonferroni’s test, 3 h: p=0.003 ).


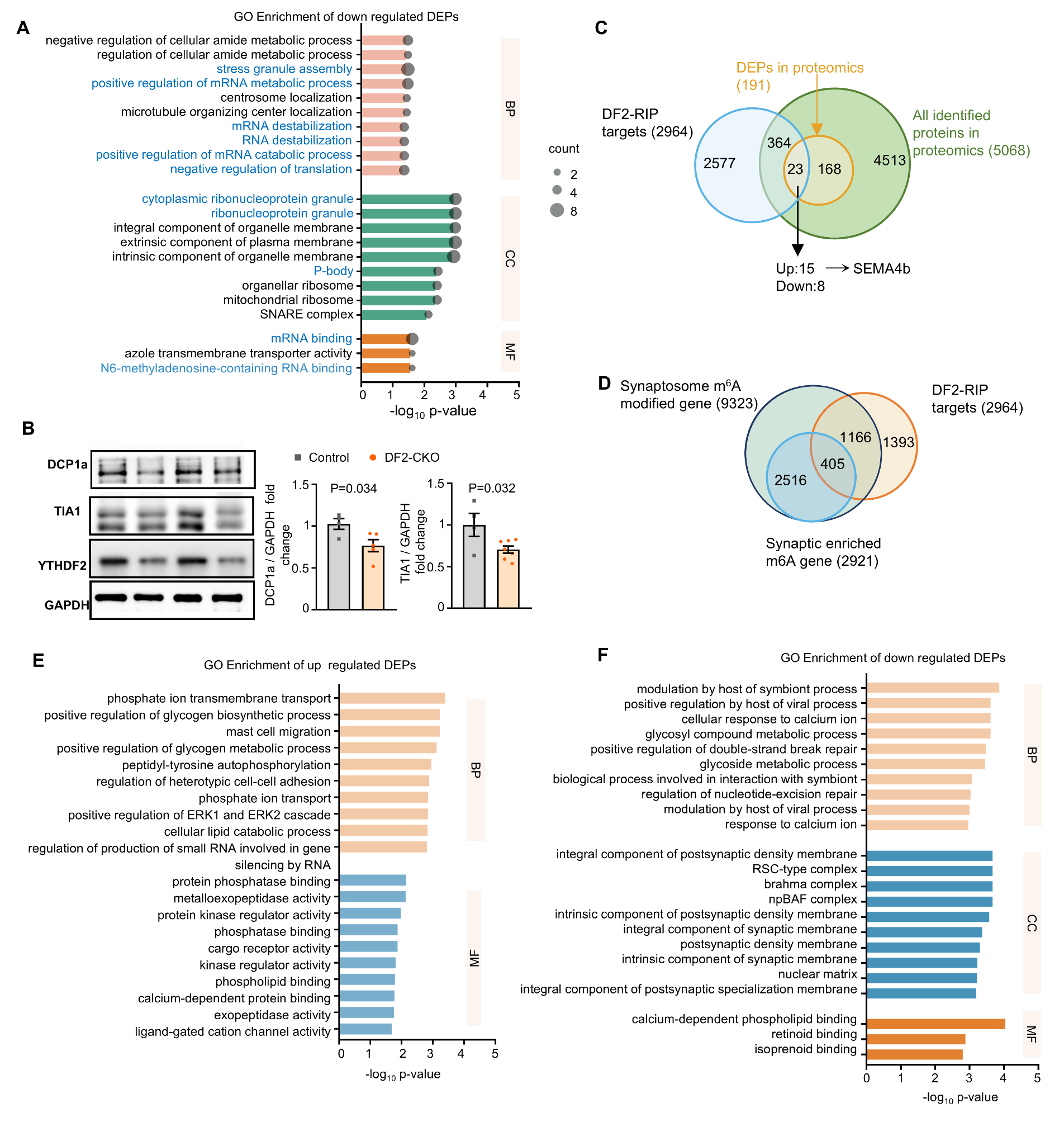


**Supplementary Figure 9. Proteomic analysis of DF2-CKO mice**

(A) GO enrichment analysis was conducted on the downregulated proteins from hippocampal proteomics at 4 h after fear conditioning. Top ten BP terms, CC terms, and the significant MF terms were showed. Downregulated protein was defined as foldchange (DF-CKO/ Control) <0.83 and p <0.05.

(B) Western blot analysis was performed to verify the expression of DCP1a and TIA1. Representative blots (left) and quantification data (right) showed increased DCP1a and TIA1 expression in hippocampus protein of DF2-CKO mice at 4 h after fear conditioning. (DCP1a: n = 4, 5 mice, unpaired two-tailed *t* test, t_7_ = 2.620, p = 0.034; TIA1: n = 4, 7mice, unpaired two-tailed *t* test, t_9_ = 2.539, p = 0.032). DCP1a: a marker of P-body; TIA1: a marker of stress granule.

(C) A Venn diagram illustrated the overlap between YTHDF2 RIP targets and the differentially expressed proteins (DEPs) identified in hippocampal proteomics. Among the 23 DEPs identified, 15 were upregulated, and 8 were downregulated.

(D) A Venn diagram illustrated the overlap between YTHDF2 RIP targets and the m^6^A-modified synaptic transcriptome (as previously reported^1^). The synaptosome m^6^A-modified genes refer to those with detected m^6^A peaks in forebrain synaptosomes, while synaptic-enriched m^6^A genes are defined as those with enriched m^6^A peaks in synaptosomes compared to homogenate lysates^1^.


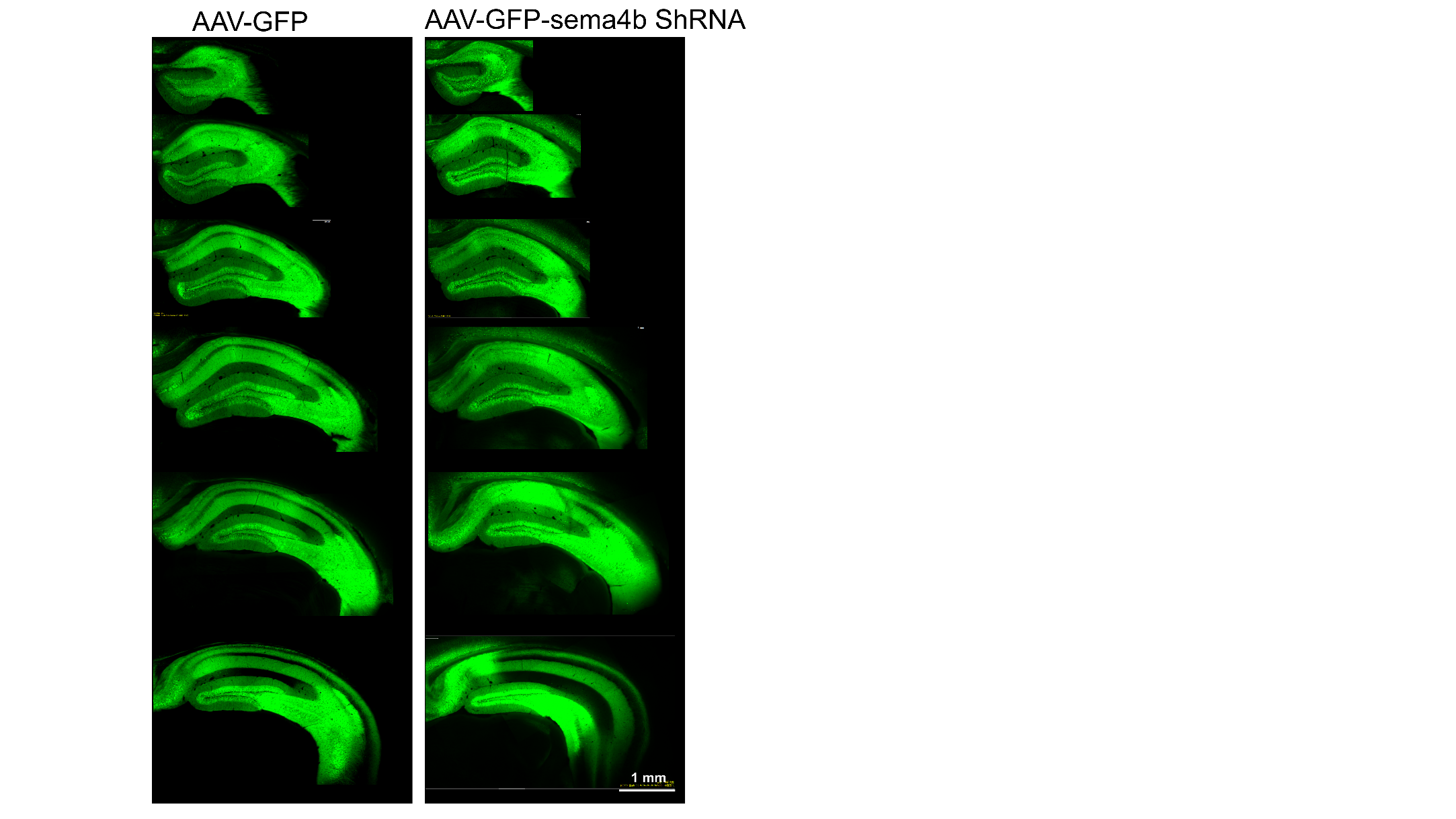


**Supplementary Figure 10. Expression of AAV-GFP-Sema4b ShRNA.**

Representative fluorescent images of AAV-GFP-Sema4b ShRNA/AAV- GFP. The observed fluorescence signal indicates widespread expression of *Sema4b* ShRNA throughout the hippocampus.

**Reference**

1 Merkurjev, D. *et al.* Synaptic N(6)-methyladenosine (m(6)A) epitranscriptome reveals functional partitioning of localized transcripts. *Nat Neurosci* **21**, 1004-1014, doi:10.1038/s41593-018-0173-6 (2018).
